# Reactive vapor-phase dealloying-alloying turns oxides into sustainable bulk nano-structured porous alloys

**DOI:** 10.1126/sciadv.ads2140

**Published:** 2024-12-18

**Authors:** Shaolou Wei, Yan Ma, Dierk Raabe

**Affiliations:** ^1^ Max-Planck Institute for Sustainable Materials, Max-Planck-Straße 1, Düsseldorf 40237, Germany.; ^2^ Delft University of Technology, 2,2628 CD Delft, Netherlands.

## Abstract

For millennia, alloying has been the greatest gift from metallurgy to humankind: a process of mixing elements, propelling our society from the Bronze Age to the Space Age. Dealloying, by contrast, acts like a penalty: a corrosive counteracting process of selectively removing elements from alloys or compounds, degrading their structural integrity over time. We show that when these two opposite metallurgical processes meet in a reactive vapor environment, profound sustainable alloy design opportunities become accessible, enabling bulk nanostructured porous alloys directly from oxides, with zero carbon footprint. We introduce thermodynamically well-grounded treasure maps that turn the intuitive opposition between alloying and dealloying into harmony, facilitating a quantitative approach to navigate synthesis in such an immense design space. We demonstrate this alloy design paradigm by synthesizing nanostructured Fe-Ni-N porous martensitic alloys fully from oxides in a single solid-state process step and substantiating the critical kinetic processes responsible for the desired microstructure.

## Teaser

A thermodynamically guided alloy synthesis paradigm directly turns oxides into sustainable bulk nanostructured porous alloys.

## INTRODUCTION

**Fig. 1.**
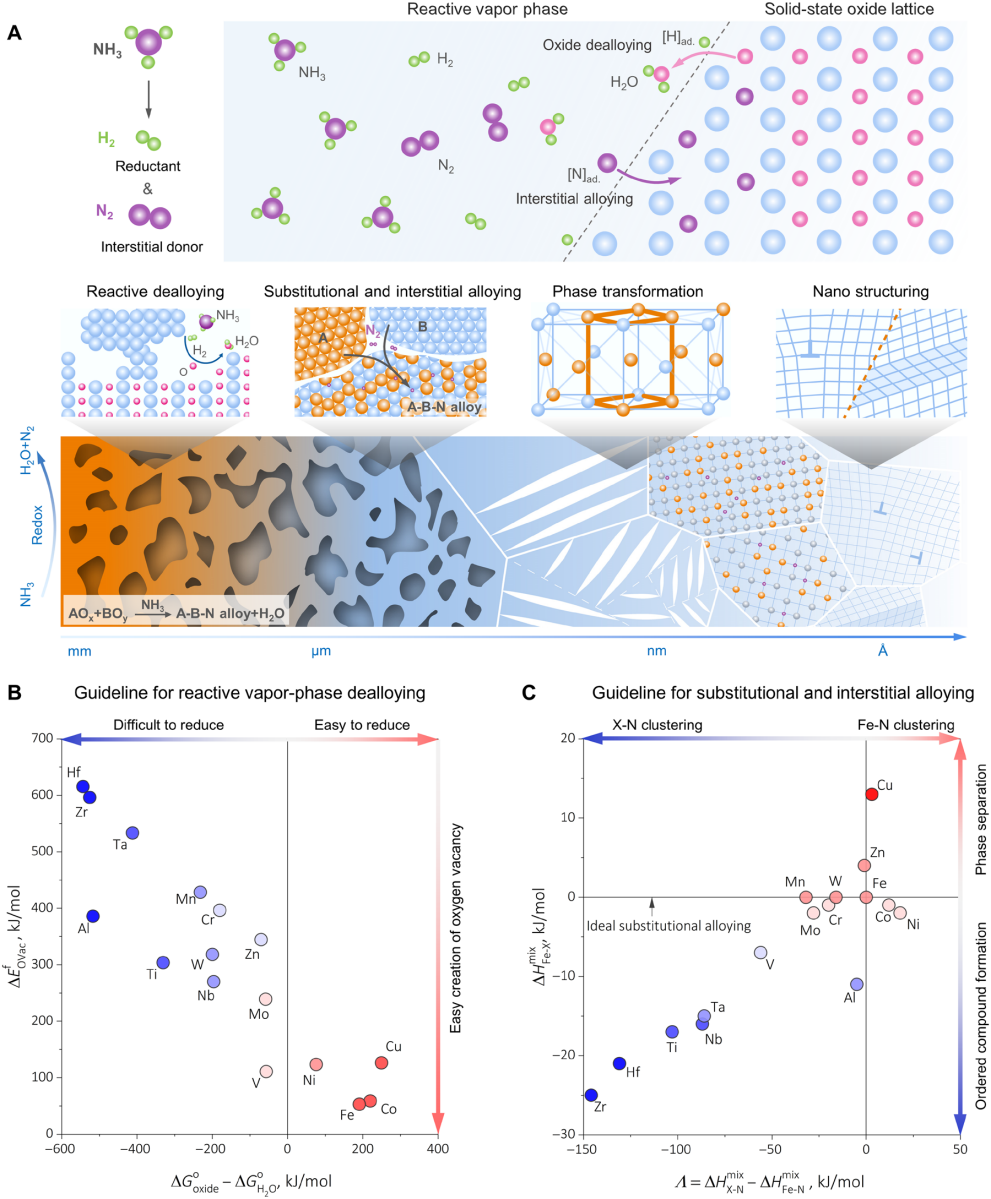
Thermodynamics-based microstructural design guidelines for reactive vapor-phase dealloying-alloying synthesis. (**A**) A sketch of the alloy design framework along with multiscale microstructural evolution microevents, encompassing reactive dealloying, substitutional alloying, interstitial alloying, phase transformations, and nanostructuring. The sketch shows the case of NH_3_ as reaction partner for the oxides, providing H_2_ as the reductant and N as the interstitial alloying element. (**B**) First thermodynamic treasure map that assesses bulk oxide reducibility and the easiness of oxygen vacancy creation at the atomic level, guiding reactive dealloying. For simplicity, the $∆G_{\text{oxide}}^{o}-∆G_{H_{2}O}^{o}$ values were calculated at 700°C, 1 atm condition for oxides with the highest valence states, using the Scientific Group Thermodata Europe (SGTE) thermodynamic database (*48*) and the literature results (*6*, *49*). The $∆E_{\text{OVac}}^{f}$ datum points were collected from the recent ground state density functional theory–based simulations (*50*–*58*). Complementary thermodynamic considerations of the vapor pressures of pure metallic elements and the boiling points of oxides are further summarized in fig. S1. (**C**) Second thermodynamic treasure map that quantifies the alloying capability, assuming Fe as the solvent and N as the interstitial solute. $∆H_{\text{Fe}-X}^{\text{mix}}$, the mixing enthalpy between Fe and the substitutional alloying element X, and the $\Lambda =∆H_{X-N}^{\text{mix}}-∆H_{\text{Fe}-N}^{\text{mix}}$ values are both computed using the literature data based on the semi-empirical Miedema model (*59*). Physical rationale of the Λ parameters is discussed in greater depth in note S1.

Since their birth through alloying, almost all metallic materials have faced a common adversary: dealloying. Dealloying is a corrosive mass transport process that selectively removes the most active constituent under certain boundary conditions (*1*–*3*), causing microstructural degradation over time. The quantized unit of dealloying, individual atom removal from a pre-existing crystal lattice (*4*, *5*), however, sparks the alloy design proposition of our work: Oxygen, the most abundant alloying element in the earth’s crust and ubiquitous in natural minerals (*6*, *7*), may also be removed from a corresponding oxide lattice if it reveals a higher affinity to the vapor phase than to its solid-state host lattice (*8*). Such a process, however, is often associated with an excessive energy cost (e.g., through the Schottky reaction $\text{Base}\to V_{O}^{\cdot \cdot }+{V\prime \prime }_{M}$, where $V_{O}^{\cdot \cdot }$ stands for the positively charged oxygen vacancy and ${V\prime \prime }_{M}$ stands for the negatively charged cation vacancy, following the Kröger-Vink notation), largely due to the ionic bonding nature of the oxides. The most effective approach to overcome this intrinsic penalty is to conceive redox reactions through the vapor phase, facilitating $O_{O}^{\times }\to \frac{1}{2}O_{2}+V_{O}^{\cdot \cdot }+2e\prime$ (where $O_{O}^{\times }$ is the charge-neutral oxygen atom residing in the solid-state lattice and e′ the transferred electron; Fig. 1A, top), as has been successfully practiced for decades in tuning the ionic transport properties of functional ceramics (*9*–*11*) and in the more recent solid-state production of sustainable iron from its ores via H_2_ gas (*6*, *12*, *13*). This sort of reactive vapor-phase dealloying process phenomenologically mimics the conventional electrochemical dealloying operation (*3*, *5*, *14*) in a sense that it also encourages porous structure creation through the migration and agglomeration of oxygen vacancies ($V_{O}^{\cdot \cdot }$). On the contrary to dealloying, alloying calls for individual atom addition to a host lattice, either on the substitutional sites or on the interstitial sites. The former case, substitutional alloying, can be fulfilled if sufficient atomic scale mixing (*15*) can be concurrently activated upon or after two or more oxides completely undergo reactive vapor-phase dealloying at elevated temperatures. The reactive vapor-phase environment, on the other hand, serves as a natural repository of interstitials, through which nitrogen and carbon can dissolve into the existing alloy via gas-solid reactions (Fig. 1A, top), as supported by the fruitful investigations on gaseous nitriding or carburizing (*16*–*18*). Altogether, these considerations form the conceptual basis for our proposed reactive vapor-phase dealloying-alloying synthesis route. We will demonstrate this approach by first theoretically assessing the energetics of the critical processes involved.

## RESULTS

Starting with oxides, we aim to integrate three principal metallurgical processes in one single solid-state operation (Fig. 1A, middle): (i) excessive porosity creation through oxide dealloying [i.e., the redox-catalyzed mass loss (*19*)]; (ii) solid-state substitutional alloying via interdiffusion among metallic species; and (iii) interstitial alloying by harvesting vapor-phase constituents. The resulting microstructure is expected to be naturally hierarchical and nanostructured because alloying unlocks the metallurgical treasure of phase transformation (Fig. 1A, bottom), the most viable pathway of multiscale microstructural modulation. We ground our a priori theoretical guideline in thermodynamics and examine the energetics for the critical reactive dealloying-alloying phenomena that are involved. Figure 1B reveals the first thermodynamic treasure map that assesses the feasibility of reactive dealloying, exploiting two physical parameters: (i) $∆G_{\text{oxide}}^{o}-∆G_{H_{2}O}^{o}$, which quantifies the bulk reducibility of oxides when H_2_ serves as the predominant reducing agent, according to the Ellingham-Richardson diagram (*6*, *20*) and (ii) $∆E_{\text{OVac}}^{f}$, the oxygen vacancy ($V_{O}^{\cdot \cdot }$) formation energy, which describes the easiness of creating the quantized unit of reactive dealloying, since at the atomic scale, our conceived process closely follows: $O_{O}^{\times }\to \frac{1}{2}O_{2}+V_{O}^{\cdot \cdot }+2e\prime$. Desirable consistency is seen between the bulk reducibility and the energetics of $V_{O}^{\cdot \cdot }$ formation, identifying Fe, Ni, Co, and Cu as the promising metallic elements that can be fully obtained by dealloying oxygen from their oxides with the highest valence states.

To synthesize any bulk alloy, sufficient atomic-scale mixing among alloying elements is indispensable, calling the need for a second thermodynamic treasure map that quantifies alloying capability. Motivated by a carbon-free alloy design framework that exploits NH_3_ as the reducing agent (*21*, *22*), we thus conceptualize substitutional and interstitial alloying by presuming Fe as the solvent and nitrogen (N) as the interstitial solute and opting for two physical parameters (Fig. 1C): (i) $∆H_{\text{Fe}-X}^{\text{mix}}$, the mixing enthalpy between Fe and the substitutional solute element X and (ii) $\Lambda =∆H_{X-N}^{\text{mix}}-∆H_{\text{Fe}-N}^{\text{mix}}$, the mixing enthalpy distinction between X-N and Fe-N that quantifies the propensity of N clustering or nitride formation. The detailed physical foundations of the Λ parameter are rationalized in note S1, following the Bragg-Williams mean-field approach (*23*). Alloy compositions residing close to the Λ=0 and the $∆H_{\text{Fe}-X}^{\text{mix}}=0$ lines in Fig. 1C are hence likely to form uniform Fe-X-N solid solutions. The larger positive deviation to the $∆H_{\text{Fe}-X}^{\text{mix}}=0$ line indicates the stronger tendency of Fe-X phase separation and vice versa for Fe-X long-range ordered compound formation. Similarly, the larger deviation to the Λ=0 line implies the more prominent N clustering or nitride formation. In fig. S1, we further clarify the potential role of vapor pressure, concluding that the sublimation propensity of Fe, Cu, Ni, and Co is negligible below 800°C, 1 atm. Fig. 1 (B and C) together with fig. S1 serves as the theoretical basis for our reactive vapor-phase dealloying-alloying synthesis route, which could inspire an immense number of alloy compositions, synthesized in a single step and under potentially even exothermic conditions. To unambiguously verify this alloy design paradigm, we choose to synthesize a porous Fe-10Ni-N atomic % (at %) martensitic alloy using NH_3_ as the reducing agent, aiming to demonstrate the four principal microstructural evolution microevents proposed, encompassing reactive dealloying, substitutional and interstitial alloying, phase transformations, and nanostructuring (Fig. 1A). As sketched in Fig. 1A, here, the role of NH_3_ is twofold: first, providing the hydrogen to dealloy and hence reduce the oxides, producing H_2_O as the only byproduct; and second, offering the nitrogen as interstitial alloying element, diversifying solid-state alloy design.

**Fig. 2.**
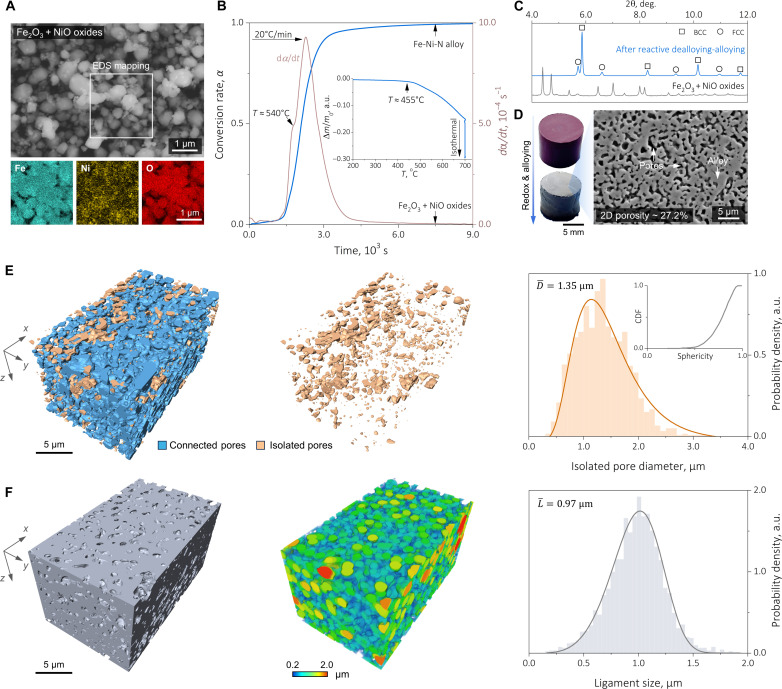
Synthesis kinetics and three-dimensional nature of the porous alloy fabricated from Fe_2_O_3_ and NiO oxides. (**A**) SE micrograph and EDS maps for the Fe_2_O_3_ and NiO mixture. (**B**) Kinetic assessment for the synthesis process. Here, the global conversion rate α(t) has been calculated from the corresponding instantaneous mass loss, as described in the Materials and Methods. The dα/dt curve is also determined, signifying that the activation of more than one reactive dealloying microevent and the inset of (B) shows that the mass loss incepts at ~455°C. (**C**) Ex situ SXRD profiles of the synthesized alloy and the mixed oxides. Only metallic FCC and BCC phases are present in the alloy specimen, and no diffraction signals from retained oxides are present. (**D**) Macroscopic images of the synthesized bulk alloy and its oxide counterpart (left). The representative low-magnification SE micrograph (right) evidences the excessive porosity within the bulk alloy. (**E**) FIB-SEM–based tomography explorations of the pores. Left: The overall three-dimensional morphology of both the connected and the isolated pores. Middle: Isolated pores. Right: Statistical analysis of the isolated pore size (D) distribution, which follows a lognormal function: $F\left(D\right)=\frac{1}{\mathrm{wD}\sqrt{2\pi }}\text{exp}\left\{-{\left[\text{ln}\left(D/\bar{D}\right)\right]}^{2}/2w^{2}\right\}$, where $\bar{D}$ denotes the mean isolated pore diameter. (**F**) Tomographic reconstruction of the alloy ligament. Left: The overall morphology. Middle: The computed ligament size. Right: The ligament size (L) distribution, as analyzed from a Weibull probability density function: $W\left(L\right)=\frac{\kappa }{L}{\left(\frac{L}{\lambda }\right)}^{\kappa -1}\text{exp}{\left(-\frac{L}{\lambda }\right)}^{\kappa }$, where κ and λ are the shape factor and the scaling factor, respectively. The mean ligament size, $\bar{L}$, is determined from $\bar{L}=\lambda \Gamma \left(1+\frac{1}{\kappa }\right)$, where Γ(x) is the gamma function: $\Gamma \left(x\right)=\int_{0}^{+\infty }\tau^{x-1}e^{-\tau }\mathrm{d\tau}$. a.u., arbitrary units.

Our synthesis starts with blending together Fe_2_O_3_ and NiO oxides and cold-compacting them into bulk pellets, targeting an Fe-10 at % Ni stoichiometry. Secondary electron (SE) micrograph along with energy-dispersive x-ray spectroscopy (EDS) maps cross-validate the uniformity of the oxide mixture (Fig. 2A) where no traits of mechanical alloying are observed. Upon heating in an NH_3_ atmosphere (Fig. 2B), the pellet starts to exhibit a discernible mass loss at ~455°C, signifying the inception of reactive dealloying, which aligns with the NH_3_ decomposition complete temperature in the literature (*22*, *24*). The corresponding conversion rate (α) shows a sigmodal-like increasing trend as the reaction progresses, which eventually plateaus at unity, confirming the complete oxygen removal from the oxides. A predominant peak accompanied by a subtle inflection is seen in the dα/dt curve, implying stepwise micromechanisms of the underlying redox reactions, which will be explored later. The synthesized bulk alloy reveals a silvery metallic appearance, contrasting the hematite red of its oxide counterpart, and ex situ synchrotron x-ray diffraction (SXRD) results further evidence the presence of metallic face-centered cubic (FCC) and body-centered cubic (BCC) phases, with no detectable retained oxides (Fig. 2, C and D, left). Lattice constants of the FCC and the BCC phases are determined through Rietveld refinement as 3.60 and 2.87 Å, respectively. Even accounting for the uncertainties involved in different measurement techniques (*25*, *26*), these values are rationally larger than those of pure Ni (~3.52 Å) and pure Fe (~2.86 Å), serving as the first hint of substitutional alloying, which results from atomic scale mixing between Fe and Ni during synthesis (*27*). While bulk in its nature, our synthesized alloy is also excessively porous: A representative cross-sectional SE micrograph reveals a two-dimensional porosity of more than 27% with worm-like alloy ligaments populating throughout the microstructure (Fig. 2D, right). These observations unambiguously support that the immense redox-catalyzed mass loss can be exploited to achieve bulk porous alloys, giving rise to reactive dealloying, as motivated in Fig. 1 (A and B).

Focusing on the pores, we next performed focused-ion beam (FIB)–SEM–based tomography measurement to explore their three-dimensional characteristics. The reconstructed tomography micrographs confirm that the pores and the alloy ligaments are indeed interpenetrating in three dimensions (movie S1), giving rise to bi-continuous morphologies [Fig. 2, E (left) and F (left)] (*28*, *29*), in which the overall volumetric porosity reaches 28.9% (Fig. 2E, left). Further connectivity quantification suggests that 94.1% of the pores are connected, while only 5.9% of them are isolated (Fig. 2E, middle, and movie S2). These topological characteristics closely mimic nanoporous metals fabricated using the conventional chemical/electrochemical dealloying routes (*5*, *14*). In addition, the massive, connected pores also hint at an evident kinetic decoupling between redox-catalyzed pore creation and surface energy-driven densification/structural coarsening (*30*), benefiting from the relatively low synthesis temperature ($<0.75T_{m}$, $T_{m}$ the bulk metal melting point). The equivalent diameter distribution of the isolated pores follows a lognormal function (Fig. 2E, right), where the mean diameter $\bar{D}=1.35\;\mu m$. The distribution of the alloy ligament size (Fig. 2F, middle), on the other hand, well aligns with a Weibull probability density function (*31*), and the mean ligament size $\bar{L}=0.97\;\mu m$ (Fig. 2F, right). The fine ligament size together with the immense porosity is suggestive of prominent grain size refinement because of the Zener-like pinning effect (*32*), which motivates dedicated microstructural explorations detailed next, starting with the validation of substitutional alloying (Fig. 1, A and C).

**Fig. 3.**
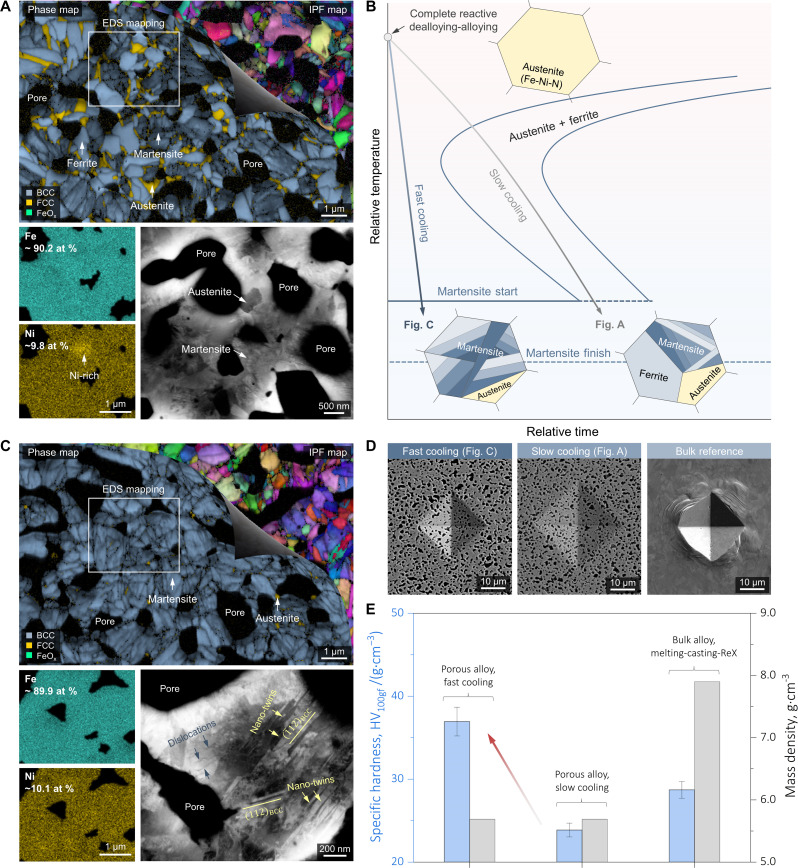
Meso-scale microstructural analyses of the synthesized bulk nanostructured porous alloys. (**A**) Microstructural validation of substitutional alloying. Top: Overlapped EBSD phase map and IPF map showing the presence of austenite, ferrite, and martensite. Bottom left: EDS maps taken across multiple grains confirming the overall spatially uniform distribution of Fe and Ni and the presence of local Ni enrichment at the austenite site. Bottom right: ECCI micrograph revealing the different defect densities in the austenite and the martensite. (**B**) Schematic of phase transformation pathway design. The three-phase microstructure in (A) suggests the possibility of achieving martensitic porous alloys by promoting the cooling rate. (**C**) Microstructure of the porous martensitic alloy following the conceived phase transformation pathway in (B) (top), overlapped EBSD phase map and IPF map revealing the martensitic microstructure (multiple variants have been nucleated) with negligible retained austenite. Bottom left: EDS maps confirming the uniform distributions of Fe and Ni at the meso-scale. Bottom right: High-magnification ECCI micrograph validates the nanostructures within the martensite, in which a high number density of nano twins and dislocations are resolved. (**D** and **E**) Specific Vickers hardness and bulk mass density measurements of the martensitic porous alloy achieved from fast cooling [left of (D)], the prototypical three-phase porous alloy [middle of (D)], and the reference Fe-10 at % Ni bulk alloy produced via the conventional melting-casting-recrystallization (ReX) route. More than seven Vickers hardness values were measured from each sample, and the error bars in (E) represent the SDs.

Figure 3A showcases the coupled electron backscatter diffraction (EBSD)–EDS analyses of the as-synthesized porous alloys where a noticeably refined microstructure is evidenced: Various submicrometer grains span over the alloy ligaments. An overall spatially homogenous distribution of Fe and Ni is seen across multiple grains (Fig. 3A, bottom left), directly confirming our conceived substitutional alloying process and the validity of the thermodynamic treasure map (Fig. 1C). A closer inspection at the EBSD phase map and the inverse pole figure (IPF) next concludes the presence of three phases, including the austenite (FCC, slightly enriched in Ni; Fig. 3A, bottom left), the nearly defect-free ferrite (BCC), and the highly-defected martensite. The presence of the martensite is rationalized by the reduced image quality in the phase map (Fig. 3A, top) (*33*), the high local kernel average misorientation value (fig. S2), and the high defect density (Fig. 3A, bottom right). These observations fully confirm our alloy design concept that phase transformations are indeed unlocked by alloying (Fig. 1A).

We note that the current microstructure achieved is to solely prove the design principle. That being stated, a broad spectrum of microstructural states is easily accessible, as sketched in Fig. 3B, by fine-tuning the phase transformation pathways. As a simple demonstrator case, we exemplify in Fig. 3C the synthesis of a light-weight porous martensitic alloy, only by promoting the post-synthesis cooling rate with the aid of air quench. In distinctive contrast to Fig. 3A, the entire microstructure almost fully consists of martensite, holding a Kurdjumov-Sachs orientation relationship (*34*) with its vicinal retained (parent) austenite (fig. S3), whose fraction barely exceeds 1.5% (prior austenite grain size is only ~1.28 μm; fig. S4). As documented in the Wechsler-Lieberman-Read theory of martensite crystallography (*35*), such a phase is intrinsically nanostructured, largely due to the pronounced lattice invariant shear deformation involved. Our high-magnification electron channeling contrast imaging (ECCI) micrograph further verifies this (Fig. 3C, bottom right): A high number density of $\left\{11,\bar{2}\right\}\langle 111\rangle$ nano twins are seen (20 to 30 nm in thickness each) within the martensite accompanied by dislocations. Benefiting from the eminent defect strengthening contribution (*36*), the porous martensitic alloy reveals a salient advantage in specific hardness ($36.9\;{\text{HV}}_{100\text{gf}}\cdot g^{-1}\cdot {\text{cm}}^{3}$) over the as-synthesized porous alloy ($23.9\;{\text{HV}}_{100\text{gf}}\cdot g^{-1}\cdot {\text{cm}}^{3}$) and its bulk Fe-10 at % Ni counterpart ($28.7\;{\text{HV}}_{100\text{gf}}\cdot g^{-1}\cdot {\text{cm}}^{3}$) while maintaining a low mass density of only 5.69 g/cm^3^ (Fig. 3, D and E). Given the high specific hardness, the porous martensitic alloy also exhibits plastic deformability as no cracking or ligament microfracture is observed at any edges of the hardness indent (Fig. 3D), motivating larger-scale high specific strength martensitic foam design as future work. While the demonstrator case discussed here in Fig. 3 primarily targets miscible solid solution Fe-Ni-N porous alloys, in figs. S5 and S6, we further validate our thermodynamic treasure map by demonstrating the synthesis of nitride-containing Fe-N and phase-separating Fe-Cu-N porous alloys.

**Fig. 4.**
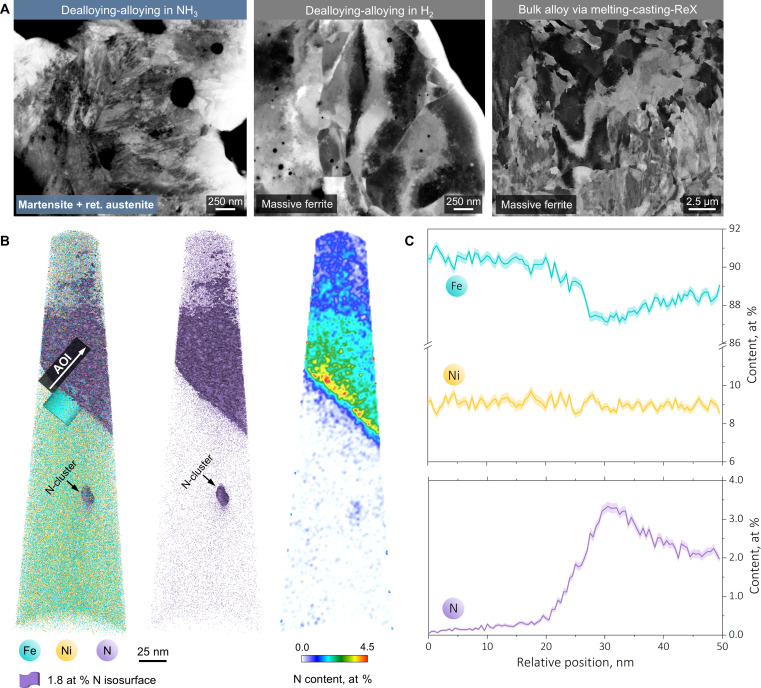
Atomic-scale evidence of N interstitial alloying. (**A**) ECCI micrographs of three alloys with the same substitutional alloying element content (Fe–10 at % Ni) but processed using different methods. Left and middle: Dealloying-alloying synthesis with the reactive vapor phase, respectively, chosen as pure NH_3_ and pure H_2_. Right: Conventional melting-casting-recrystallization. It is seen that the desired martensitic microstructure is only available when NH_3_ serves as the vapor-phase constituent, and the other two alloys both exhibit massive ferrite-like microstructures despite the cooling rates are kept identical in all three cases. (**B**) Three-dimensional APT measurements validating both Fe-Ni substitutional alloying and N interstitial alloying. Left: The overlapped Fe, Ni, and N distributions with an 1.8 at % N isosurface highlighted. Middle: N distribution in which a nano-sized N cluster is also present (also see fig. S8 for complementary analyses). Right: Two-dimensional contour map of the N content, revealing the segregation possibly at planar defect sites within the martensite. (**C**) One-dimensional Fe, Ni, and N distribution profiles acquired from the cylindrical area of interest sketched in (B).

Coming back to the overall alloy design scheme (Fig. 1), the only remaining pillar that necessities substantiation is interstitial alloying. We propose that the presence of martensitic transformation discussed in Fig. 3 already serves as meso-scale evidence: Such a beneficial phase transformation is only accessible when the porous alloys are synthesized in NH_3_ (Fig. 4A, left), where N interstitials can be harvested from the vapor phase, contributing to tetragonal distortion of the martensite (*37*, *38*). This principle can be easily generalized (see Fig. 1A, top) as also other types of martensite can be synthesized by exploiting alternative reactive vapor-phase constitution (e.g., CH_4_, C_2_H_2_ or even their mixtures) that can dealloy oxygen and simultaneously donating an interstitial alloying element capable of triggering martensitic transformations. By contrast, a porous alloy obtained when using H_2_ alone as the reducing agent (see fig. S7 for quantitative kinetic comparisons) and its bulk counterpart fabricated through the conventional melting-casting-recrystallization route both exhibit massive ferrite-like microstructures (Fig. 4A, middle and right) (*39*, *40*), even when the global Ni content and the cooling method were kept identical.

Zooming into the atomic scale, the presence of N interstitial [~0.41 at % for the entire atom probe tomography (APT) tip] is unequivocally confirmed by three-dimensional APT measurements (Fig. 4B, left). N also reveals a certain segregation tendency presumably at the planar defect sites within or at the vicinity of the martensite (Fig. 4B, middle and right), where the local N content reaches up to ~3.0 at %, according to a cylindrical area of interest quantification (Fig. 4C and also see fig. S8 for complementary analyses). This sort of spatially inhomogeneous distribution of N introduces an additional ingredient to our alloy design scheme (Fig. 1A), through segregation engineering (*41*), a well-recognized physical metallurgy approach to tailor the microstructure-property synergy. To ensure the reproducibility of the APT measurements, we have also carried out a separate set of measurements (fig. S9), in which the similar characteristics of N spatial distribution are also observed.

## DISCUSSION

**Fig. 5.**
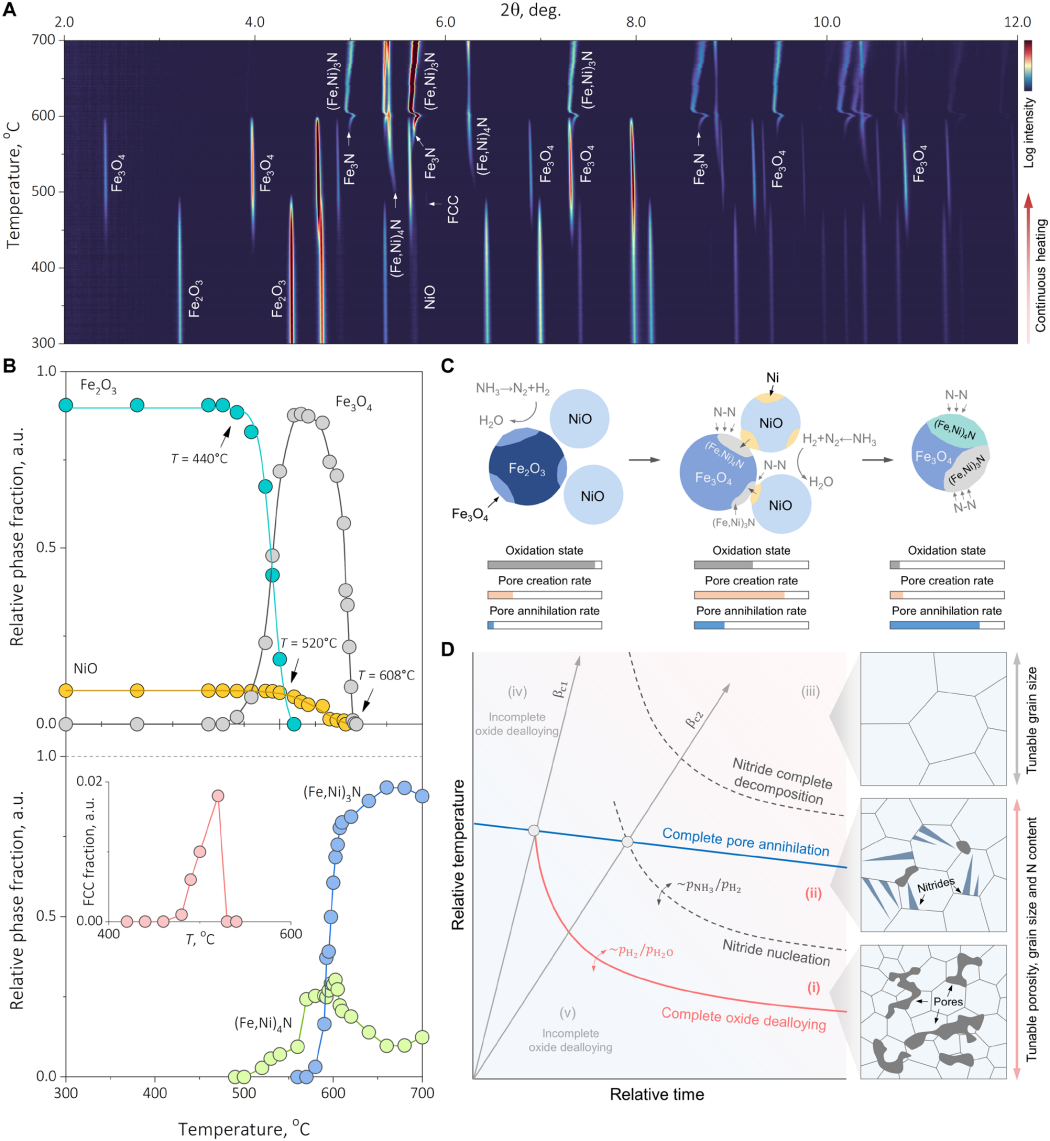
In situ synchrotron x-ray explorations of the synthesis mechanisms and a kinetic conception for microstructural design. (**A**) Two-dimensional contour map revealing the progression of the dealloying-alloying process from which oxide dealloying and different nitrides are evidenced. (**B**) Relative phase fraction change as a function of temperature, quantified through Rietveld refinement. The relative phase fraction change of NiO and Fe_2_O_3_ over temperature aligns with the Boltzmann’s sigmoidal function: $f\left(T\right)=f_{f}+\left(f_{i}-f_{f}\right){\left[1+\text{exp}\left(\frac{T-T_{p}}{dT}\right)\right]}^{-1}$, where $T_{p}$ denotes the temperature when f=0.5 (489°C for Fe_2_O_3_ and 557°C for NiO), dT is the rate factor (9.1 for Fe_2_O_3_ and 23.3 for NiO), and $f_{i}$ and $f_{f}$ are the phase fractions at the initial and the final stage, respectively. We note that because all the redox reactions involve mass loss, and the phase fraction of H_2_O is unmeasurable via in situ SXRD, datum points in (B) cannot be used to back-calculate the absolute phase fraction or establish quantitative correlation with the thermogravimetry measurements. (**C**) Schematic of the synthesis mechanisms and the involvement of nitrides. Here, the extents of oxidation state, pore creation rate, and pore annihilation rate are also qualitatively sketched at various stages of the reaction. (**D**) Ashby-type kinetic conception map qualitatively outlining the accessible microstructures. Regions (i) and (ii) reveal the kinetic window where tunable porosity, grain size, and different nitride states are possible. Regions (iii) represents salient microstructural coarsening after complete nitride decomposition with fully densified microstructures. Regions (iv) and (v) denote incomplete oxide dealloying where immense amounts of oxides might still be retained in the microstructures.

Having elaborated the possibility of harvesting interstitial N from the vapor phase (Fig. 4B), we emphasize that our proposed alloy design scheme may go far beyond the prototypical scope of solid solution alloy synthesis (Fig. 1C), through which multiple metallic nitrides are also accessible (also supported by the presence of nitrides in figs. S5 and S6). To substantiate this point and to also shed more quantitative light on the operating reactive dealloying-alloying micromechanisms, we opt for in situ SXRD measurements (details of the experimental instrument are provided in fig. S10), aiming to elaborate phase constitution change over time (movie S3). The progression of the reactions is visualized in Fig. 5A, where stepwise redox pathways are evident, rationalizing the multiple inflection points seen in the global dα/dt curve (Fig. 2B). By Rietveld refining the time-resolved diffractograms, it is recognized that the leading redox reaction Fe_2_O_3_ → Fe_3_O_4_ is activated at ~440°C, followed by the NiO → Ni step, initiating at ~520°C (Fig. 5B and also see Fig. 5C as a guide to the eye). As a result, the relative phase fractions of Fe_2_O_3_ and NiO monotonically decrease, respectively diminishing at ~520° and ~590°C, and their kinetics well complies the Boltzmann’s sigmoidal function (*42*). The nonmonotonic evolution observed in the Fe_3_O_4_ phase fraction, which peaks at ~0.87 in the 520–560°C temperature range, quantitatively confirms its presence as the intermediate step of the entire dealloying-alloying process. A minor transient metallic FCC phase (maximally ~0.02) is only momentarily present in the 480°–530°C temperature range (Fig. 5, B and C), cross supporting the inception of substitutional alloying discussed in Figs. 1 and 3. This phase is further identified as pure Ni via lattice constant analysis, as revealed in fig. S11. Two predominant nitride phases, the γ′-(Fe,Ni)_4_N and the ε-Fe_3_N/(Fe,Ni)_3_N, respectively, start to nucleate at ~500° and ~580°C, achieving final equilibrium fractions of 0.48 and 0.52 during prolonged isothermal holding at 700°C for 1800 s (fig. S12). In note S2, we also provide detailed rationalization for the unequivocal involvement of Ni substitutional alloying in driving phase transformations within the nitrides.

All the foregoing observations and analyses not only validate the applicability and usefulness of the thermodynamic treasure maps proposed in Fig. 1 (B and C) but also allow us to derive an associated plausible Ashby-type of kinetic design guideline, as sketched in Fig. 5D, qualitatively considering the interplays among (i) reactive oxide dealloying, (ii) pore creation/annihilation, and (iii) nitride nucleation/decomposition, which are the three most predominant mass transport micromechanisms identified earlier. Regions (i) and (ii) bounded by the oxide dealloying complete line (tunable via the redox potential (*20*); $p_{H_{2}}/p_{H_{2}O}$) and the complete pore annihilation line represent the most viable kinetic windows where tunable porosity and grain size are accessible. The dashed nitride nucleation line, which is also controllable by the nitriding potential (*16*) $\left(p_{NH_{3}}/p_{H_{2}}\right)$ serves as a switch to modulate between solid solution and nitride-containing microstructures. In contrast, process parameters surpassing the complete pore annihilation and the complete nitride decomposition lines [region (iii)] will instead only enable tunable grain size within the fully densified microstructures. Although any quantitatively precise construction of such a kinetic treasure map still necessities extensive future efforts, the significance of heating rate can still be discussed, for which we hypothesize two critical values, $\beta_{c1}$ and $\beta_{c2}$, based on the microstructure states at the complete pore annihilation temperatures. The former represents the maximum possible heating rate for complete oxide dealloying, and the latter indicates the minimum heating rate to pursue full solid solution microstructures. While the current kinetic conception emphasizes heating and isothermal holding, presuming an infinitely fast cooling rate and ideal stability of the microstructure acquired at elevated temperatures, some of the metastable nitride phases might be more easily accessible through implementing a fine-tuned cooling trajectory to diversify the phase transformation pathways via solid-state nitriding.

In summary, we reform the intuitive opposition between alloying and dealloying in metallurgical processing and introduce here an oxide-oriented metallic alloy design paradigm that encompasses vapor-phase reactive dealloying, substitutional alloying, interstitial alloying, phase transformations, and nanostructuring in one solid-state operation. Such an approach assigns all atoms from both reaction partners specific roles: H_2_ from the NH_3_ for oxygen removal in the reactive dealloying step and N_2_ for providing interstitials in the alloying step. We propose thermodynamic treasure maps that can quantitatively navigate the alloy synthesis in the profound composition-processing space and exemplify the synthesis of Fe-Ni-N bulk nanostructured porous martensitic alloys completely from oxides. Through dedicated multiscale microstructural analyses and in situ diffractometry explorations of the governing redox and alloying micromechanisms, we also introduce an Ashby-type kinetic concept that unlocks microstructural design opportunities. The universality of our approach goes beyond the specific scope of porous solid solution synthesis yet can be readily extended to: (i) bulk metallic nitride permanent magnetic alloys and (ii) alloy foam fabrication from oxide contaminated feedstocks or metallurgical wastes. Our alloy design paradigm also inspires future synergetic efforts among chemical synthesis, extractive, and physical metallurgy to turn oxides (or even minerals) directly into application-worthy products through diverse reactive vapor phases.

## MATERIALS AND METHODS

### Alloy synthesis

The raw materials exploited in the synthesis were standard 325-mesh Fe_2_O_3_, NiO, and Cu_2_O oxide powders with >99% purity, which closely mimics purified natural ores or minerals with all gangue oxides removed. Low-energy ball mixing (250 rpm for 5 hours with a ball to material ration of 5:1) was adopted to mix the powders following the chemical stoichiometry of the conceived metallic alloys (Fe-10Ni and Fe-10Cu, at % in the present work). No discernible mechanical alloying has taken place during the mixing process, as evidenced by the ex situ SXRD measurements (details revealed in the proceeding section). The mixed powders were cold compacted into green pellets with ~13-mm diameter and ~3-mm thickness using a commercial hydraulic pressing instrument. Each individual pellet was kept to ~2.5 g to ensure consistent mass-loss kinetic measurements and to mitigate possible sample size effect during the synthesis. No cold isostatic pressing was involved in the present study, while such a process step is recommended for practical application-oriented future work where scalability serves as the primary consideration.

The synthesis along with the kinetic measurements was carried out in an in-house thermogravimetry setup equipped with a temperature-programmed infrared heating device. To minimize potential fluctuations in the mass balance during the measurement, the specimen was stabilized in NH_3_ (5 liter/hour; purity of both is greater than 99.999%) flow for 10 hours before each test. A moderate heating rate of 20°C/min was chosen for the synthesis. The pellet specimen was heated up in NH_3_ gas flow (10 liter/hour) up to 700°C, followed by a 2.5-hour isothermal holding period to encourage complete oxide reduction, during which the instantaneous mass was continuously recorded with a datum acquisition rate of 1 Hz. Upon completing the isothermal holding period, the infrared furnace was fully shut down to enable a cooling rate of approximately 120°C/min. The instantaneous dimensionless conversion rate α(t) of the oxide reduction process was then quantified as: $\alpha \left(t\right)=\left[m_{0}-m\left(t\right)\right]/\left(m_{0}-m_{\infty }\right)$, in which $m_{0}$ and m(t) are the initial mass and the instantaneous mass of the pellet specimen, respectively. $m_{\infty }$ denotes the theoretical final mass, which was obtained by assuming complete oxide-to-metal conversion for individual oxide within the pellet, i.e., Fe_2_O_3_ → Fe, NiO → Ni, and Cu_2_O → Cu. To assess the role of nitrogen interstitial alloying when using NH_3_ as the vapor-phase redox reaction agent, similar synthesis routes were also carried out in pure H_2_, for which the experimental details have been kept consistent.

### Multiscale microstructural characterization

The synthesized specimens were subjected to microstructural analyses, aiming to uncover the critical dealloying-alloying microevents at multiple length scales. Phase constitution was first analyzed by ex situ SXRD, carried out at beamlines P02.1 and P21.2, PETRA III of Deutsches Elektronen-Synchrotron (DESY). Two-dimensional diffractograms were collected using a 60-keV high-energy x-ray beam (correspond to a wavelength of 0.20735 Å) with a size of 0.5 mm by 0.5 mm and an exposure time of 10 s. The specimen-to-detector distance was optimized as ~960 mm, which enables a desired balance between the spatial resolution and the number of accessible Debye-Scherrer diffraction rings. The instrumental parameters were also calibrated with a NIST-standard LaB_6_ powder. Phase fraction and lattice constant were obtained by Rietveld refining the integrated diffractograms through a GSAS-II open-access software (*43*), and the residual error $R_{\text{wp}}$ was maintained below 8% for individual refinement.

Polycrystal-scale (hereafter referred to as meso-scale) analyses of the synthesized porous alloys were conducted on a Zeiss Merlin scanning electron microscope (SEM), equipped with EDS and EBSD detectors. Specimens for SEM-EDS-EBSD characterizations were prepared following the conventional metallographic routes: They were first cross-sectioned from the as-synthesized pellets using a low-speed diamond wire saw to minimize potential damage or deformation to the ligaments within the porous alloys. These cross-sectional slices were then ground on a series of SiC abrasive papers up to #1000 grit, followed by polishing using diamond suspension with 3- and 1-μm particle size. The final polishing was carried out using 40-nm particle size colloidal SiO_2_ polishing solution for ~30 min and was first cleaned with soap and subsequently acetone bath to completely remove any SiO_2_ particles that agglomerated inside the pores. Coupled EBSD and EDS maps were acquired under a working distance of 18 mm with an accelerating voltage of 15 keV and a beam current of 5 nA. The raw EBSD diffractograms were post-analyzed in an Orientation Imaging Microscopy software to obtain quantitative crystallographic information. Surface trace and martensite habit plane analyses were performed using a home-built STrCryst software package (https://github.com/shaolouwei/STrCryst), of which the detailed experimental protocols along with the limitations of the methodology has been addressed in greater depth in our previous work (*44*).

To explore the three-dimensional characteristics of the pore topology at the meso-scale, FIB-SEM–based tomography study was carried out using an FEI Helios Xe plasma FIB (PFIB) instrument. The cross-sectioned specimen was subjected to serial sectioning via PFIB with an individual slice thickness of ~50 nm. A total of 300 slices were made, translating into a total thickness of ~15 μm, which is more than an order of magnitude larger than the two-dimensional pore size (~1.2 μm), securing statistical reliability. Per each PFIB sectioning interval, a high-resolution SE micrograph (1242 × 922 pixel^2^) was taken. Image segmentation for the pores and the alloy ligament was conducted using a JAVA-based random forest classifier (*45*, *46*) that exploited the SE contrast. The final three-dimensional volumetric rendering along with further quantitative assessment were both accomplished through an Avizo software.

Zooming into the nanoscale, three-dimensional APT was exploited to evidence the presence of nitrogen interstitials in a spatially resolved manner. Lamellar lift-out and tip granular milling were performed using an FEI Helios NanoLab600i dual-beam SEM. In view of the immense porosity inside the specimen, an in situ chromium coating method (*47*) was used to further ensure the mechanical stability of the APT tip upon being extracted from the microstructure. The raw APT data were collected using a Cameca LEAP 5000XS instrument under the laser-pulsing mode at 50 K, with a laser frequence and pulse energy of 40 pJ and 200 Hz, respectively. The 3D atom maps associated with quantitative post-analysis were conducted using an AP Suite 6.1 software.

### In situ synchrotron x-ray experiment

The phase transformation mechanisms accompanied with the dealloying-alloying synthesis were investigated using in situ SXRD at P02.1 PETRA III of DESY. The same beam energy (60 keV, wavelength of 0.20735 Å) as the ex situ measurements was chosen for the in situ tests, while the specimen-to-detector distance was kept as ~1700 mm. The precompacted oxide powders were sealed in a quartz capillary tube with an inner diameter of ~500 μm, and a type-K thermocouple was place right next to the specimen for temperature measurement as the reaction progresses. The capillary tube was heated up with a proportional-integral-derivative (PID)–controlled ceramic radiation heater up to 700°C under the presence of 1 atmospheric pressure NH_3_ flow. To mitigate convection cooling, a mild flow rate of ~10 ml/min was adopted. A sketch of the experimental setup and a picture of the actual configuration of the apparatus are provided in fig. S10. During the experiment, two-dimensional diffractograms were recorded every 5 s, enabling time-resolved observation of the activation of oxide reduction and any solid-state phase transformations that might be at play. These two-dimensional raw diffractograms were first azimuthally integrated (0^o^ to 90^o^ quarter ring) using the GSAS-II open access software (*43*). Diffraction peak position shift was tracked using a pseudo-Voigt regression function, and the change in relative phase fraction was determined through Rietveld refinement ($R_{\text{wp}}<10\%$ was reached for individual refinement).

## Supplementary Materials

### The PDF file includes:

Figs. S1 to S18 Notes S1 and S2 Legends for movies S1 to S3 References
