# Supplementary material for: Reactive vapor-phase dealloying-alloying turns oxides into sustainable bulk nano-structured porous alloys
Source: Sci Adv. 2024 Dec 18;10(51):eads2140. doi: 10.1126/sciadv.ads2140 (PMC11654685; doi:10.1126/sciadv.ads2140)
Supplement: Supplementary file 1 — Figs. S1 to S18 Notes S1 and S2 Legends for movies S1 to S3 References [file sciadv.ads2140_sm.pdf]

Science Advances
AAAS

Supplementary Materials for

Reactive vapor-phase dealloying-alloying turns oxides into sustainable bulk nano-structured porous alloys

Shaolou Wei *et al.*

Corresponding author: Dierk Raabe, d.raabe@mpie.de; Shaolou Wei, sl.wei@mpie.de

Sci. Adv. **10**, eads2140 (2024)
DOI: 10.1126/sciadv.ads2140

The PDF file includes:

Figs. S1 to S18
Notes S1 and S2
Legends for movies S1 to S3
References

Other Supplementary Material for this manuscript includes the following:

Movies S1 to S3

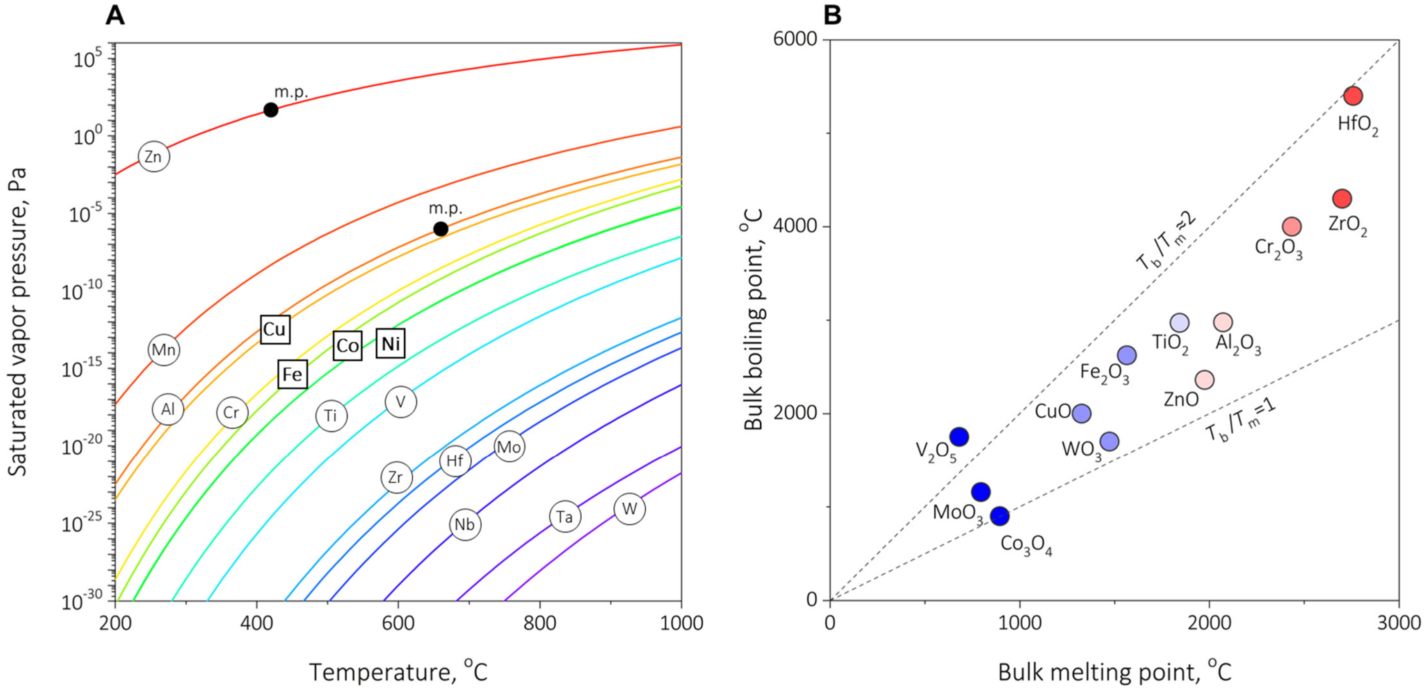

Fig. S1 | Complementary thermodynamic considerations of vapor pressure and oxide melting/boiling points. (A) Saturated vapor pressure of all the metallic elements considered in main text **Fig. 1 B** and **C**. At any temperatures below 800 ºC, only Zn and Mn exhibits somewhat sublimation tendencies. The four identified elements, Fe, Ni, Co, and Co that exhibit bulk reducibility at their oxides with the highest valence states (Fe_2O_3, NiO, CuO, and Co_3O_4) when H_2 acts as the reducing agent, all exhibit low vapor pressures at the level of ~10^{-15} Pa, indicating negligible sublimation tendencies. Results shown in **A** are adopted from Ref. (*60*). **(B)** Bulk boiling point versus bulk melting point chart for the oxides included in main text **Fig. 1 B** and **C**. It is seen that except for MoO_3 and Co_3O_4, other oxides barely melt below 1000 ºC, simultaneously revealing larger distinctions between the bulk melting and the bulk boiling temperatures. We also note that literature record of the boiling point of NiO is absent, largely because of its decomposition before reaching the boiling state. Datum points in **B** are obtained from the literature (*8*, *49*, *60*).

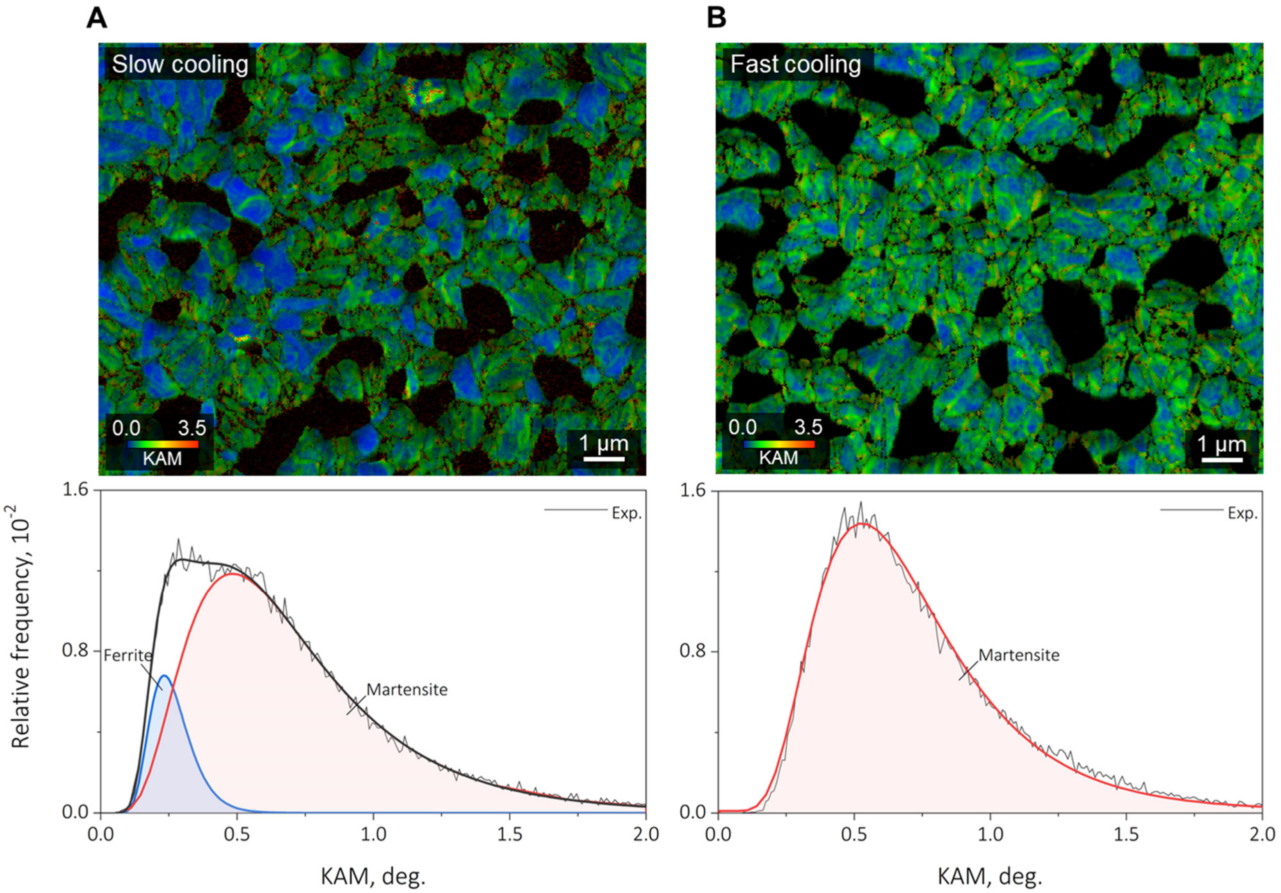

Fig. S2 | Kernel average misorientation maps for the two porous Fe-Ni-N alloys obtained with different cooling rates. (A) The slow cooling case (main text **Fig. 3 A**), where bimodal distribution in the relative frequency of the kernel average misorientation (KAM) is present, indicating the presence of both ferrite (the lower KAM phase) and martensite (the higher KAM phase (*61*)). **(B)** The fast cooling case (main text **Fig. 3 A**), where only the higher KAM martensite is present. The KAM values are obtained by considering up to the second nearest neighboring point and a misorientation tolerance of 3.5°.

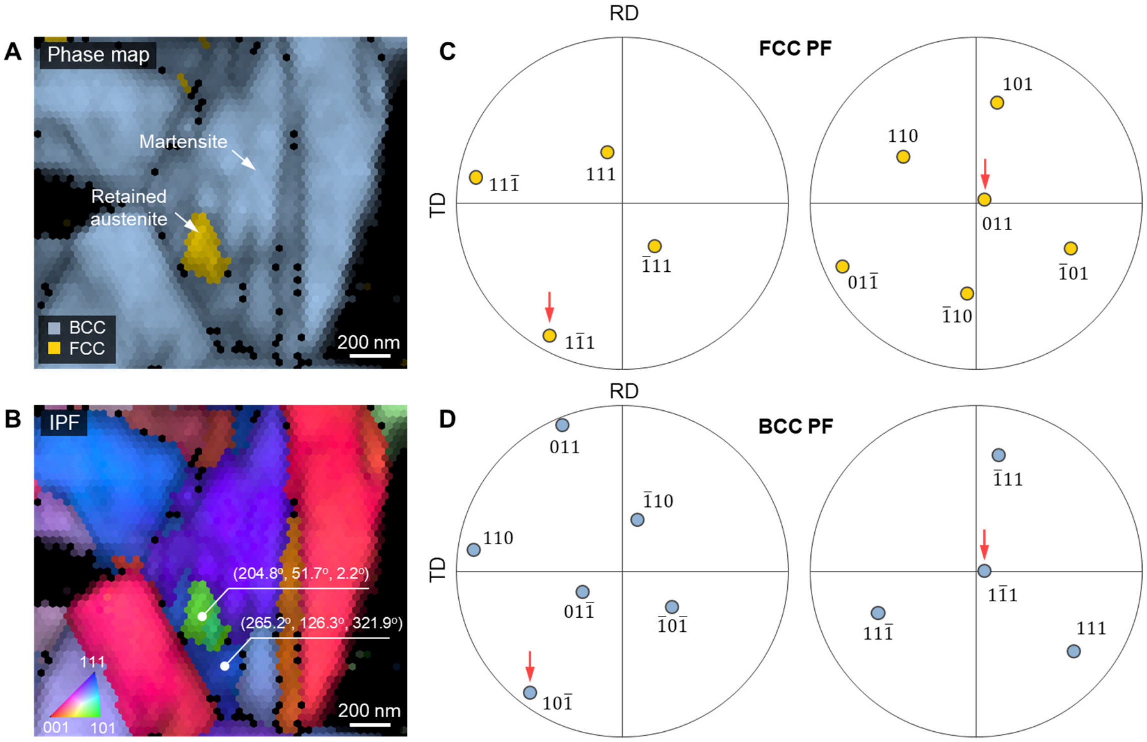

Fig. S3 | Orientation relationship between martensite and parent austenite. **(A)** EBSD phase map showing the representative region for the analysis, in which both the martensite and the retained (parent) austenite are present. **(B)** Inverse pole figure (IPF) corresponding to **A**. The Euler angles of the martensite and the retained (parent) austenite are obtained for orientation relationship analyses. **(C)** $\{111\}_{FCC}$ and $\{110\}_{FCC}$ pole figures. **(D)** $\{110\}_{BCC}$ and $\{111\}_{BCC}$ pole figures. Comparison between **C** and **D** confirms that the assessed martensite and its vicinal retained (parent) austenite exhibits a Kurdjumov-Sachs (K-S) orientation relationship: $(1\bar{1}1)_{FCC} \parallel (10\bar{1})_{BCC}$ and $[011]_{FCC} \parallel [1\bar{1}1]_{BCC}$. Such an orientation relationship has also been confirmed for more than 3 similar austenite-martensite pairs.

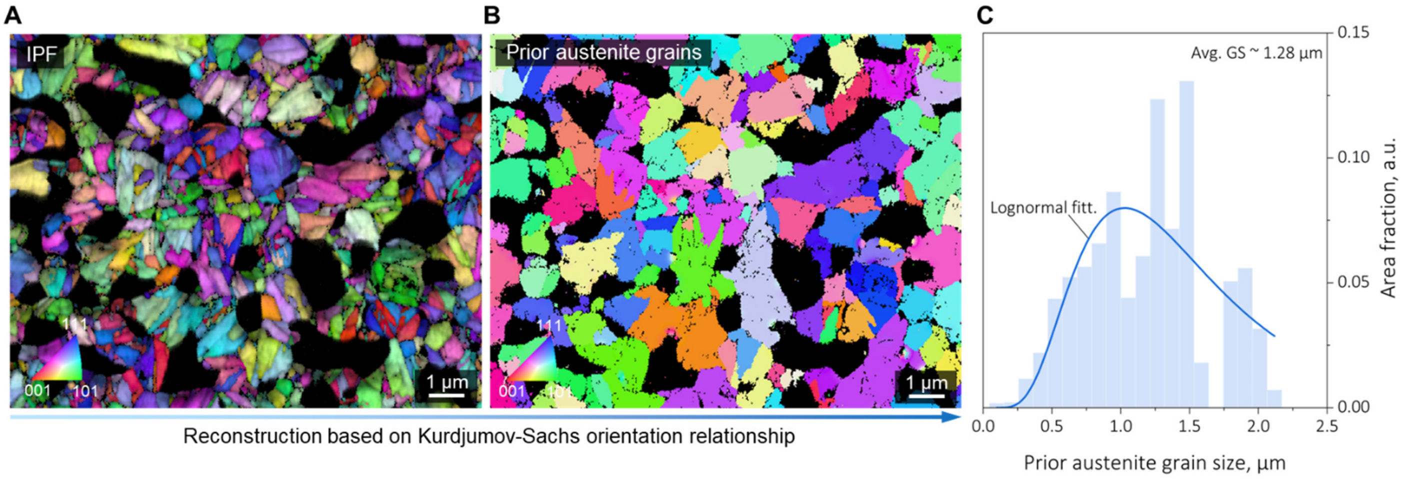

Fig. S4 | Prior austenite grain size determination. (A) IPF map corresponding to main text **Fig. 3 C** where multiple sub-micro-sized martensite variants are observed. **(B)** Reconstructed prior austenite grains using the K-S orientation relationship identified in **Fig. S3**. **(C)** Prior austenite grain size distribution where a classical lognormal trend is present with an average grain size of only ~1.28 µm. Despite such a small prior austenite grain size, the presence of multiple martensite variants suggests the predominant role of free surface nucleation *(62, 63)* which can be rationalized by the excessive porosity.

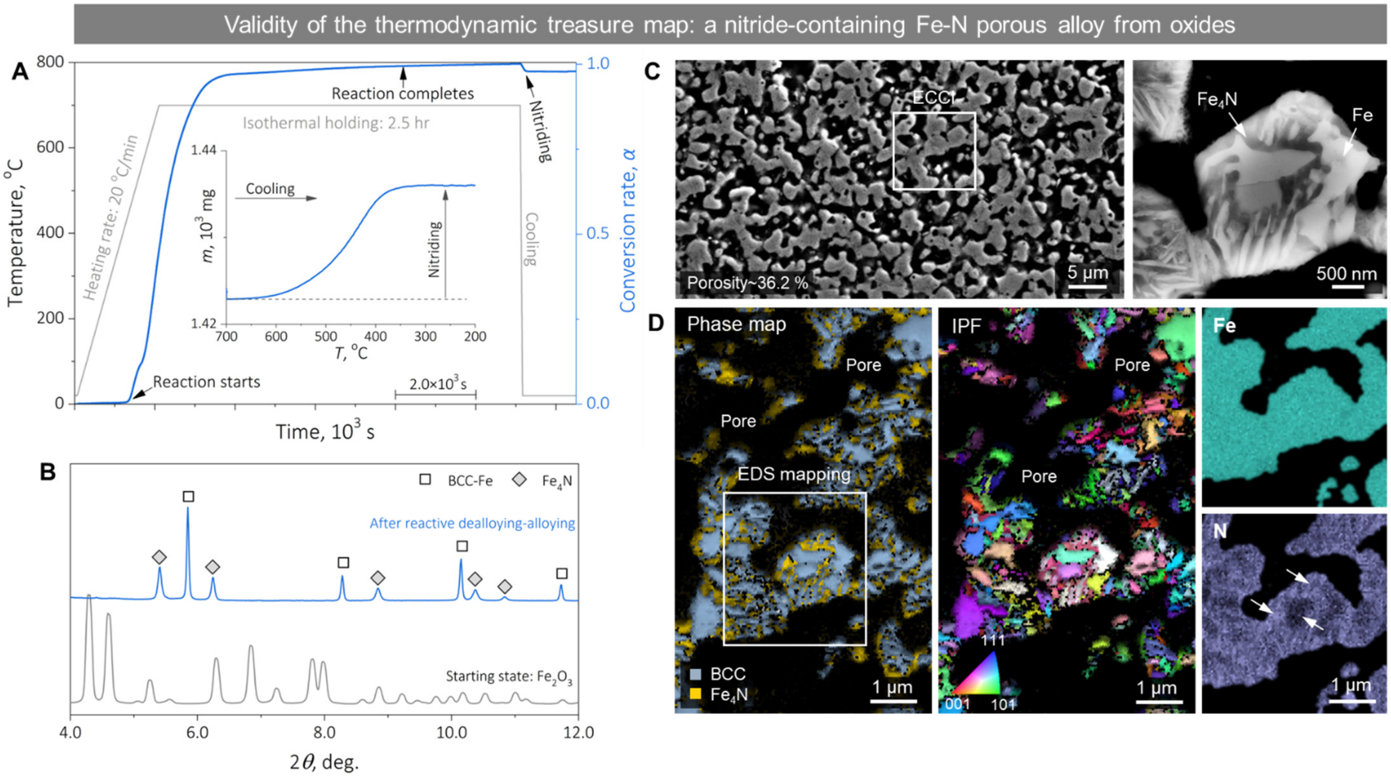

Fig. S5 | Synthesis of a nitride-containing Fe-N porous alloy via NH_3. (A) Global conversion rate (α) as a function of time, overlapped with the temperature profile. The starting state of the synthesis was pristine Fe_2O_3 green-compacted oxide pellet. Inset of **A** showcases the significant mass gain during cooling, signifying the onset of nitriding. **(B)** *Ex situ* synchrotron X-ray diffraction (SXRD) results confirming the presence of the BCC phase and the γ'-Fe_4N phase after synthesis and no retained oxide phase is present. **(C)** Scanning electron microscopy (SEM) investigations of the microstructure. Left: lower magnification secondary electron micrograph revealing the excessive porosity of ~36.2 %. Right: high magnification electron channeling contrast imaging (ECCI) micrograph showing the morphology of the BCC Fe-phase (presumably with N as interstitial solute) and the γ'-Fe_4N phase. **(D)** Coupled EBSD-EDS analyses. Left: phase map. Middle: IPF map revealing that both phases exhibit a substantially refined grain size below ~1 μm. Right: EDS maps of Fe and N taken across multiple grains, in which local N-rich regions are present, correlating with the γ'-Fe_4N phase evidenced in the phase map and by the SXRD patterns.

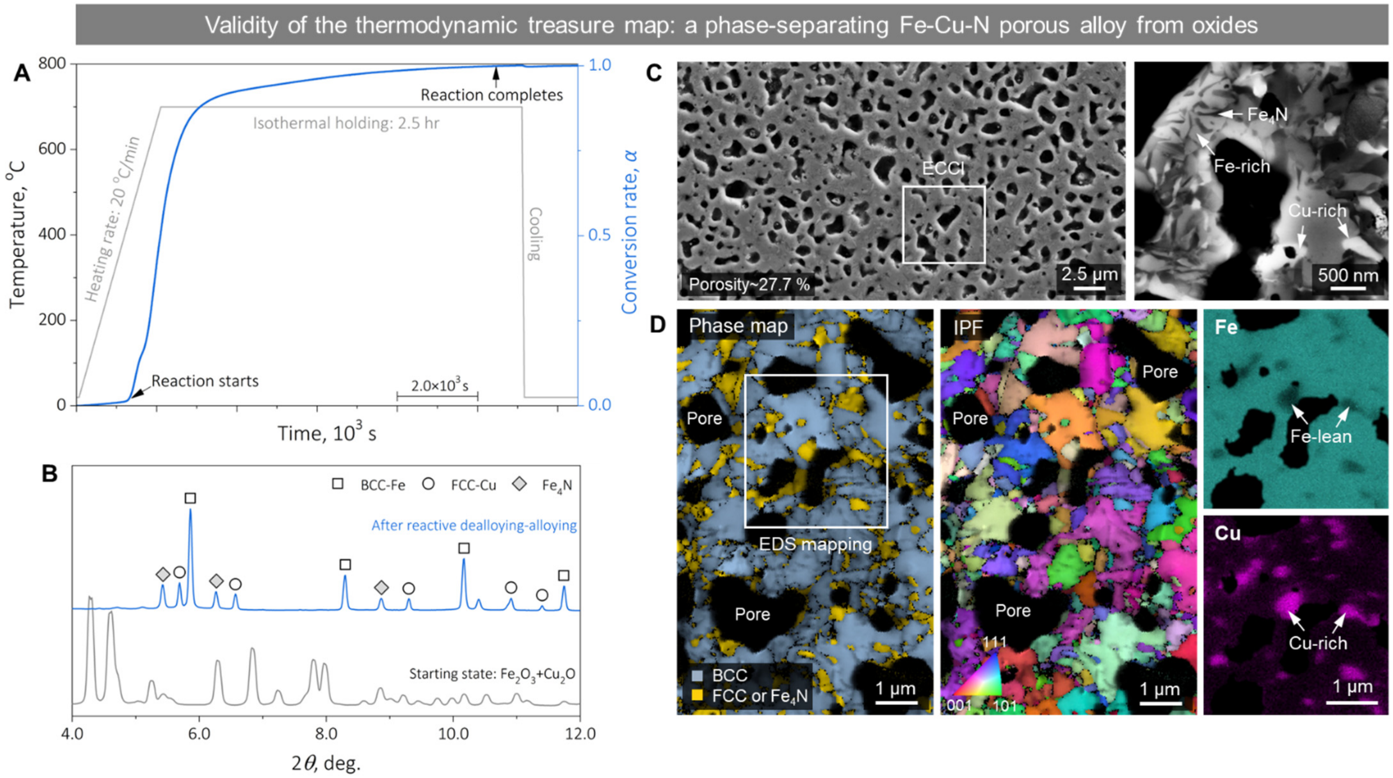

Fig. S6 | Synthesis of a phase-separating Fe-Cu-N porous alloy via NH_3. (A) Global conversion rate (α) as a function of time, overlapped with the temperature profile. The starting state of the synthesis was green-compacted Fe_2O_3 and Cu_2O mixed oxide pellet, targeting a global composition of Fe-10 at.% Cu-N, which maintains a consistent atomic fraction of substitutional alloying element as the Fe-10 at.% Ni alloy shown in the main text. **(B)** *Ex situ* synchrotron X-ray diffraction (SXRD) results confirming the presence of the BCC phase (Fe-rich), the FCC phase (Cu-rich), and the γ'-Fe_4N phase after synthesis and no retained oxide phase is present. **(C)** SEM analyses of the microstructure. Left: lower magnification secondary electron micrograph revealing the excessive porosity of ~27.7 %. Right: high magnification electron channeling contrast imaging (ECCI) micrograph showing the morphology of the BCC-structured Fe-rich phase, the FCC-structured Cu-rich phase, and the γ'-Fe_4N phase. **(D)** Coupled EBSD-EDS analyses. Left: phase map. Middle: IPF map. Right: EDS maps of Fe and Cu taken across multiple grains, where phase separation is unequivocally confirmed because of the positive mixing enthalpy between Fe and Cu ($\Delta H^{\mathrm{mix}}_{\mathrm{Fe-Cu}} = +13$ kJ/mol (*59*), also see the thermodynamic treasure map in main text **Fig. 1 C**), validating our thermodynamic *treasure map* proposed in main text **Fig. 1 B** and **C**. These observations well align with the SXRD results shown in **B**.

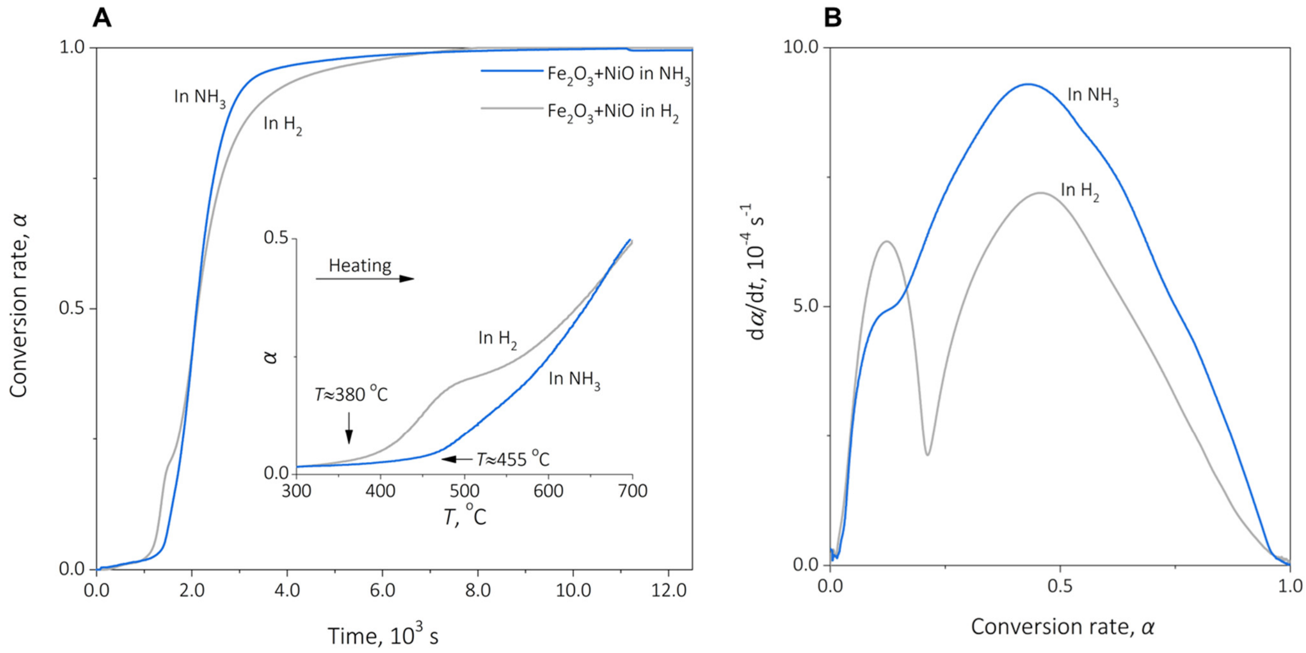

Fig. S7 | Kinetic comparison for synthesizing the same Fe-10 at.% Ni alloy using NH_3 and H_2. (A) Comparison of global conversion rate (α) as a function of time. The these two measurements, the heating rate (20 ºC/min), the green compaction condition, the target substitutional alloying element content (10 at.% Ni), and the cooling rate have all been kept identical. Inset of **A** exhibits the evolution of α over temperature, revealing that the onset of oxide dealloying in the pure H_2 atmosphere incepts at a lower temperature of ~380 ºC than that of the NH_3 case (because eminent NH_3 decomposition instead requires a higher temperature (*22*, *24*)), leading to an initially higher α below 650 ºC. An interesting cross-over point is seen in the α curve at ~670 ºC, after which the oxide dealloying speed in NH_3 becomes predominantly higher. **(B)** $\mathrm{d}\alpha/\mathrm{d}t - \alpha$ curves calculated using the results in **A**. Here two sets of peaks are evident at the similar α values of ~0.12 and ~0.42, suggesting the activation of stepwise redox micro-mechanisms, also discussed in main text **Fig. 2 B** and **Fig. 5 A**.

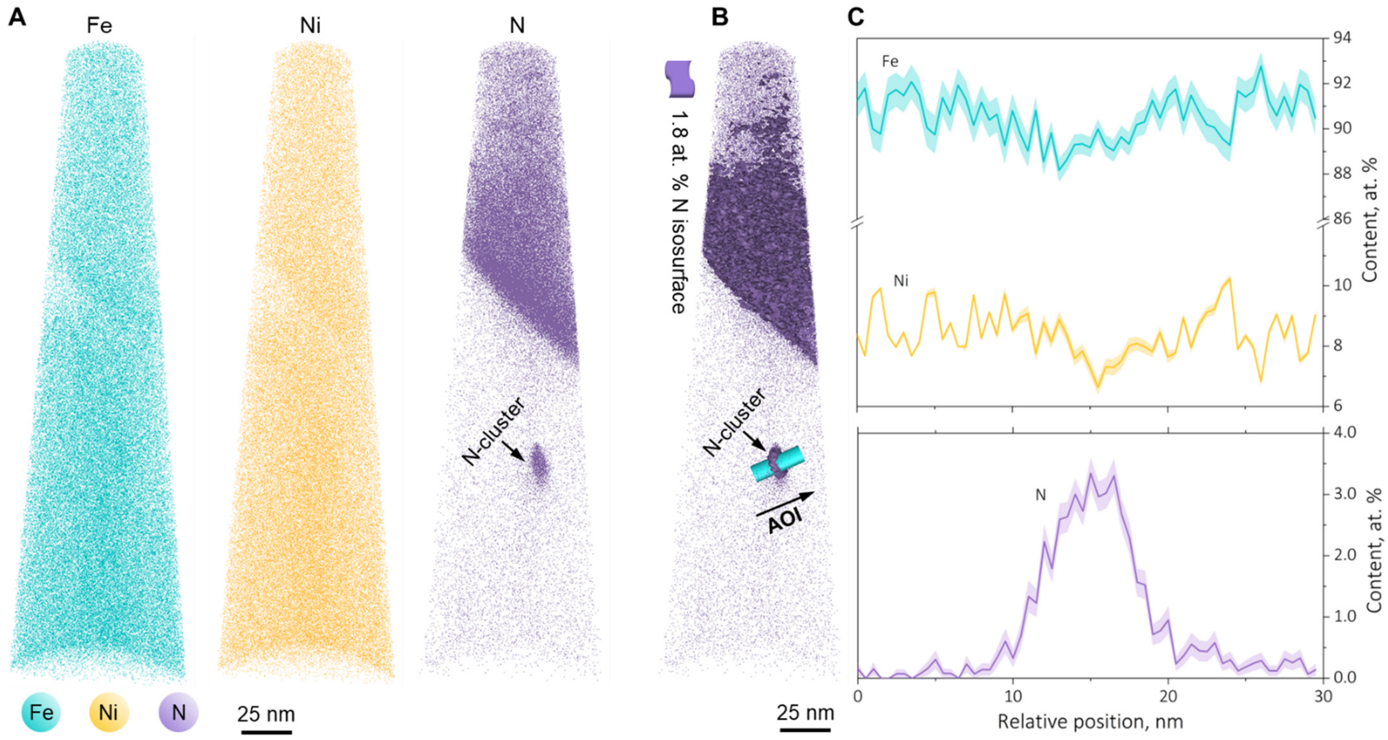

Fig. S8 | Analyses complementing the APT results revealed in main text Fig. 4. (A) Individual Fe, Ni, and N distribution showing the Fe-Ni substitutional alloying through atomic-scale mixing during the synthesis as well as the presence of N as interstitial atoms. The segregation tendency is also clear with N preferentially accumulating to the top portion of the tip and a ~10 nm-sized N cluster is also present. **(B)** Isosurface analysis of N distribution confirming the presence of N segregation, most probably at the planar defect sites within or at the vicinity the martensite plates. **(C)** One dimension distribution profiles for Fe, Ni, and N taken from the highlighted area of interest in **B**. The local N content within the clustered region can exceed ~3.0 at.%.

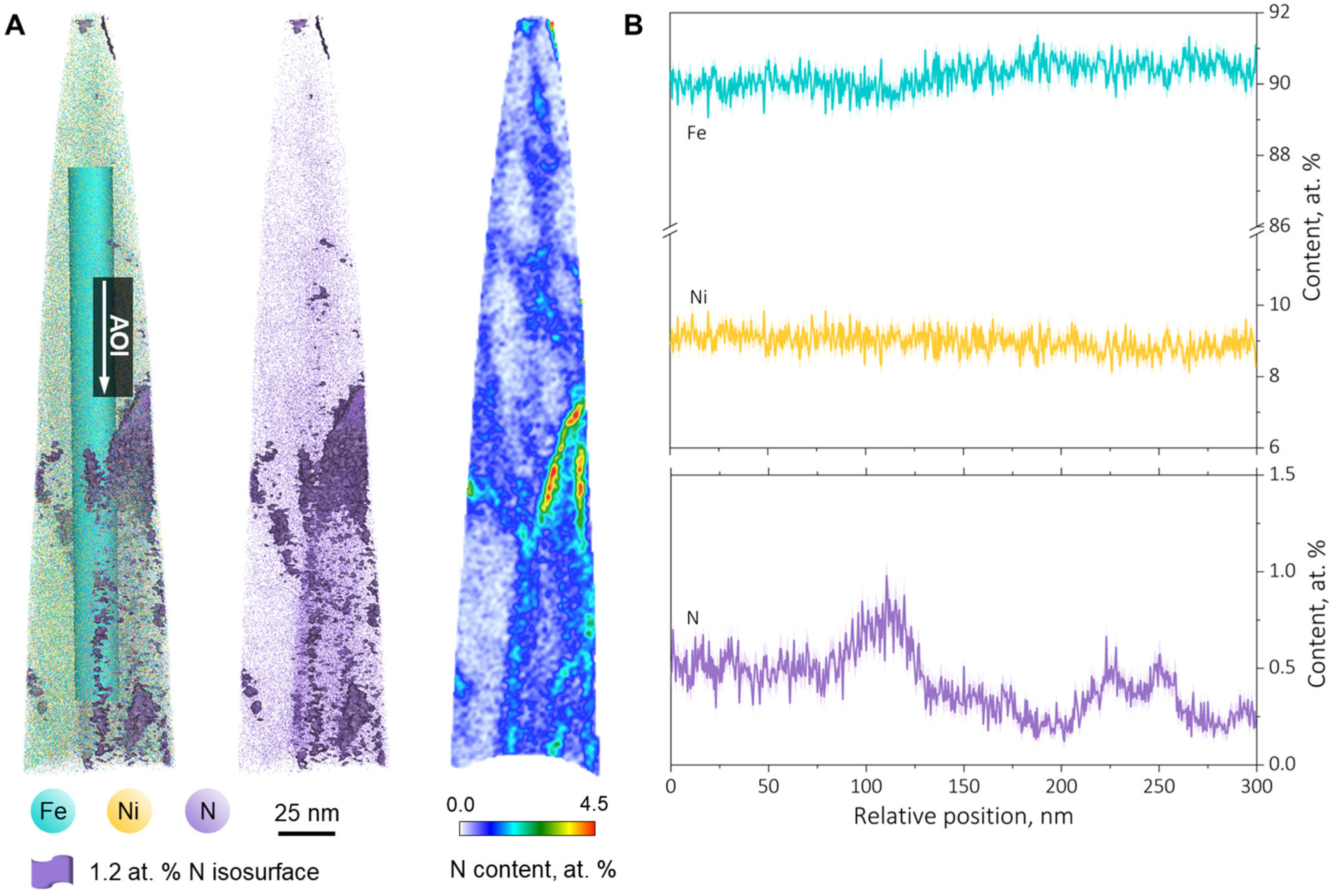

Fig. S9 | A separate set of APT measurements supporting the discussion in main text Fig. 4. (A) Three-dimensional APT measurements of Fe, Ni, and N distributions, validating both Fe-Ni substitutional alloying and N interstitial alloying. Left: the overlapped Fe, Ni, and N distributions with an 1.2 at. % N isosurface highlighted. Middle: N distribution in which the similar spatially inhomogeneous characteristics are present, as also seen in main text **Fig. 4 B**. Right: two-dimensional contour map of the N content. **(B)** One dimensional Fe, Ni, and N distribution profiles acquired from the cylindrical area of interest sketched in **A**.

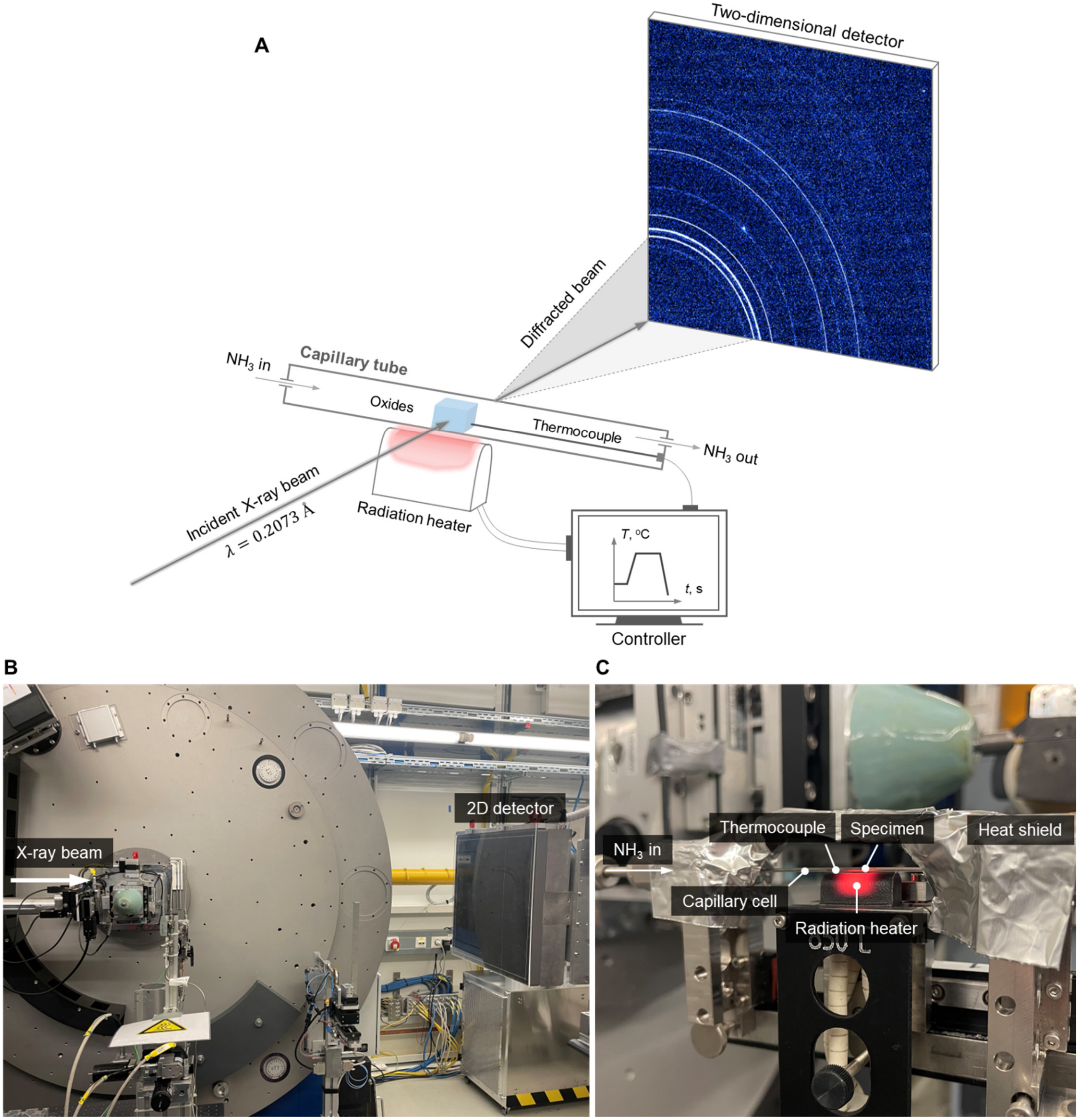

Fig. S10 | Illustration of the *in situ* synchrotron X-ray experimental setup. (A) Schematic showing the principles of the experimentation. Here the pre-compacted oxide sample is sealed in a quartz capillary cell and a type-K thermocouple is placed right next to the specimen for temperature measurements. Radiation heating is supplied from the bottom of the capillary cell during which moderate NH_3 will be flowed into the cell to activate dealloying-alloying synthesis. **(B)** An overall picture of the diffraction setup. **(C)** A zoomed-in view of the capillary cell and the radiation heating device.

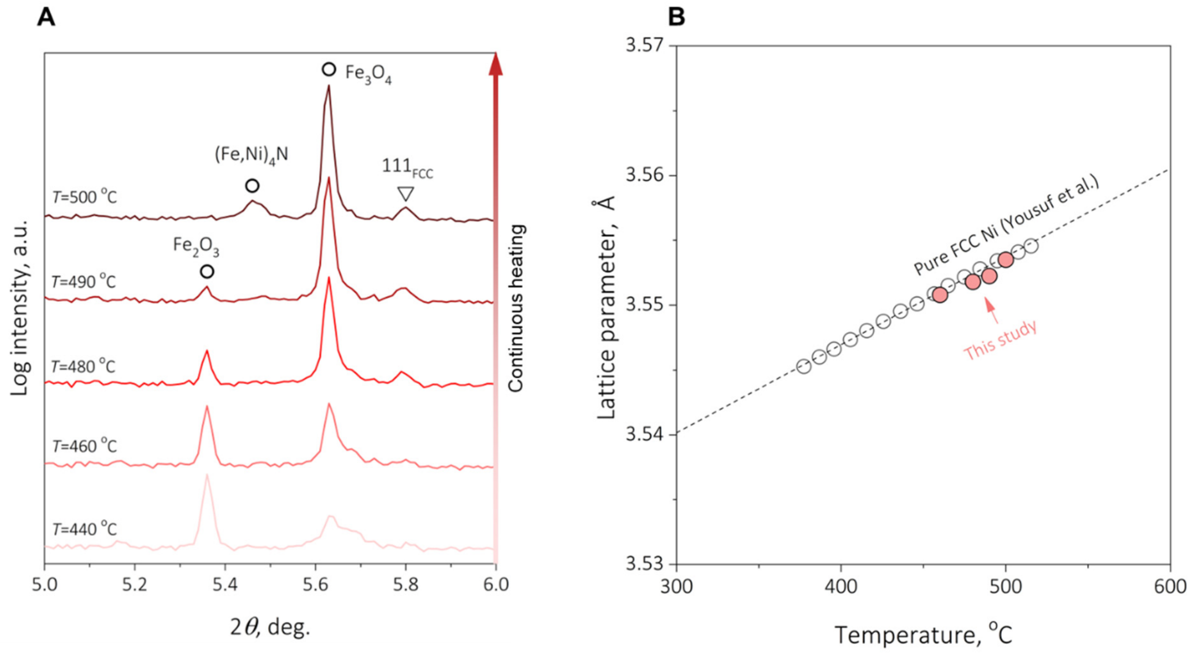

Fig. S11 | *In situ* diffraction evidence for the formation of pure Ni. (A) Representative integrated *in situ* diffractograms acquired in the temperature range of 440-500 ºC where the formation of the metallic FCC peak is unambiguously evidenced. **(B)** Lattice constant comparison between the FCC phase seen in the current study and the one reported for pure Ni by Yousuf *et al.* (*26*) Consistency is observed between these two sets of measurements, confirming that the metallic FCC phase revealed in our results is indeed pure Ni, whose phase fraction is instead minor, according to the Rietveld refinement discussed in main text **Fig. 5 B**.

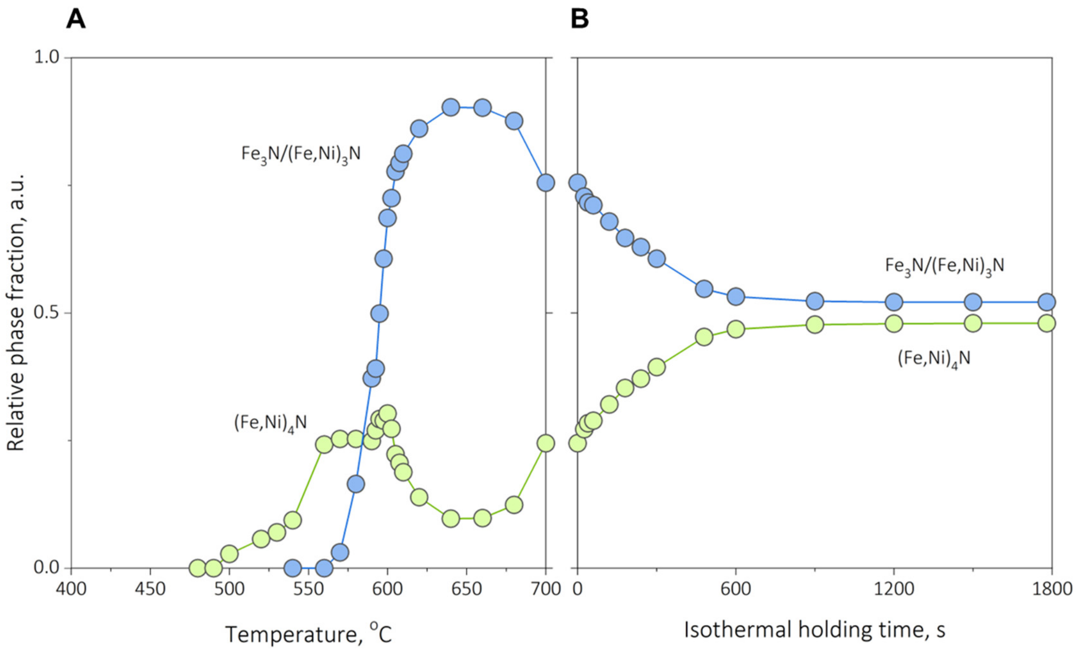

Fig. S12 | Equilibrium phase constitution at 700 ºC in the *in situ* synchrotron X-ray measurements. (A) Relative nitride phase fraction evolution as a function of temperature, reproduced from main text **Fig. 5 B. (B)** Relative phase fraction change during 1800 s isothermal holding at 700 ºC, where the ε-Fe_3N/$(Fe,Ni)_3N$ nitride phase exhibits a monotonic decreasing trend in its fraction and *vice versa* for the γ'-$(Fe,Ni)_4N$ nitride phase. At the equilibrium state these two phases respectively develop relative phase fractions of ~0.52 and ~0.48.

Note 1. Physical rationale of the thermodynamic parameter for interstitial alloying

For assessing the capability of interstitial alloying, we have introduced the thermodynamic parameter $\Lambda = \Delta H^{\mathrm{mix}}_{\mathrm{X-N}} - \Delta H^{\mathrm{mix}}_{\mathrm{Fe-N}}$ in the main text. In this ***Note***, we provide detailed physical rationalization for this parameter and discuss its further revelation for interstitial N-containing alloy design based on our proposed reactive vapor-phase dealloying-alloying paradigm (main text **Fig. 1**).

The fundamental consideration of such a thermodynamic parameter lies in the difficulty in inferring interstitial N solubility in Fe-X binary substitutional alloys from its solubility in respective pure metallic species (**Fig. S13**). It is seen in **Fig. S13 A** that the interstitial N solubility in pure metallic elements is primarily associated with the crystal structure, as historically surveyed by Goldschmidt *(64)*. Maximum N solubility in transition FCC metals is almost inversely proportional to the atomic number increase. Its solubility in transition BCC metals, on the contrary, is more closely relevant to the group number in the periodic table, *i.e.* monotonic decreasing trend is present amongst the elements locating in the same group. Immense N solubility is consistently seen in transition HCP metals, such as Ti, Zr, and Hf, while Zn appears as an exception. Comparing the results shown in **Fig. S13 A** and **B**, it is evident that N solubility in Fe-X binary substitutional alloys cannot be intuitively predicted from its respective solubility in Fe and in X, following a Vegard's law-like linear approximation *(27)*. As a simple example, N exhibits higher solubility in Co than Cr, but the exact opposite trends are seen when they form substitutional Fe-Co and Fe-Cr binary alloys. This sort of inconsistency motivates the introduction of the Λ parameter, which quantifies the interaction between Fe-N and X-N pairs in any Fe-X-N ternary alloys (**Fig. S14 A**).

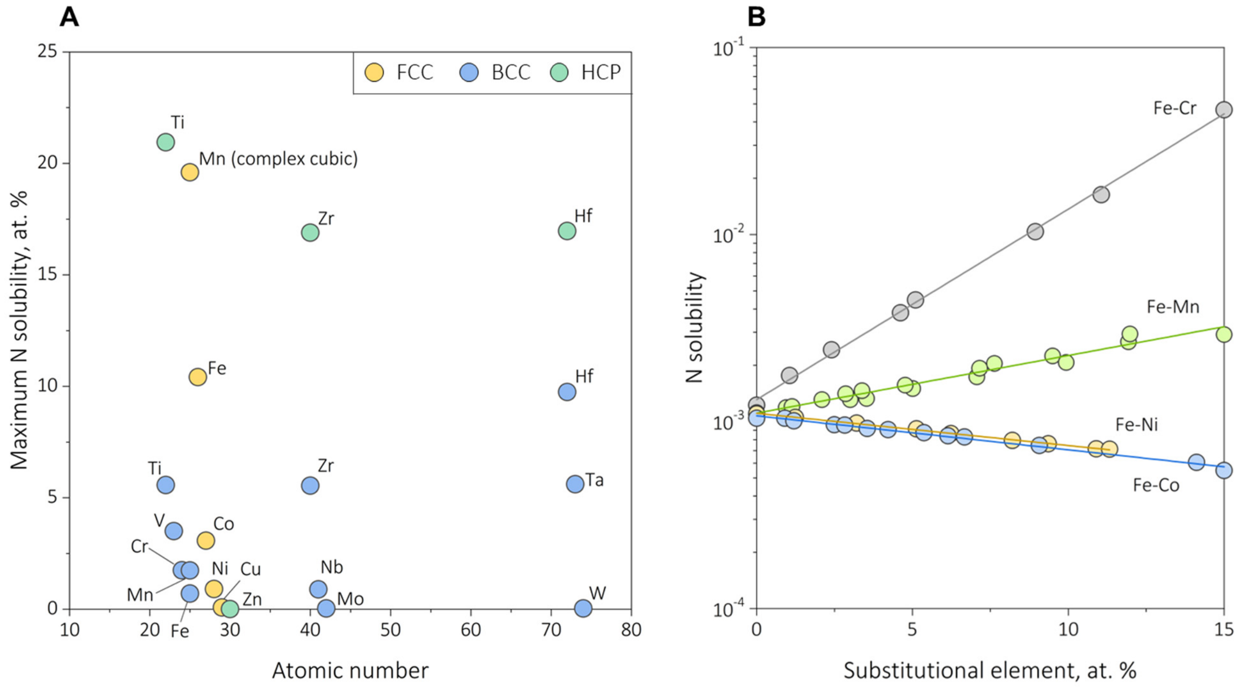

Fig. S13 | Fundamental motivations for the Λ parameter. (A) Maximum N solubility in pure transition metal elements classified by crystal structure *(64)*. **(B)** Maximum N solubility as a function of the content of substitutional alloying element in Fe-X binary alloys *(65)*.

In the following, we reveal the physical foundations of the thermodynamic parameter $\Lambda = \Delta H^{\mathrm{mix}}_{\mathrm{X-N}} - \Delta H^{\mathrm{mix}}_{\mathrm{Fe-N}}$ (**Fig. S14 A**), following a primitive Bragg-Williams mean-field approach *(20, 23)* and analytically rationalize its validity. For mathematical simplicity, we firstly introduce the following presumptions: (i) a two-dimensional simple cubic structured Fe-X binary alloy already exists prior to the introduction of any interstitial N atoms (**Fig. S14 B** and **C**); (ii) the first nearest neighbor interaction is the most predominant term in the total energetics; (iii) any quadratic fluctuations about the mean energy state of the system can be neglected; and (iv) the predominant role of configurational entropy when interstitial N atom is introduced.

Considering the interstitial alloying process sketched in **Fig. S14 C**, the total enthalpic contribution to the pre-existing Fe-X binary alloy can be approximated using the foregoing mean-field formalism:

$$\mathcal{H}_{\mathrm{N-X}} \simeq n_{\mathrm{Fe}}\varepsilon_{\mathrm{Fe-N}} + n_{\mathrm{X}}\varepsilon_{\mathrm{X-N}} = n\varepsilon_{\mathrm{Fe-N}} + n_{\mathrm{X}}(\varepsilon_{\mathrm{X-N}} - \varepsilon_{\mathrm{Fe-N}}) = n\varepsilon_{\mathrm{Fe-N}} + \Delta\varepsilon_{\mathrm{N-X}}n_{\mathrm{X}} \tag{S1}$$

Where, $\varepsilon_{\mathrm{Fe-N}}$ and $\varepsilon_{\mathrm{X-N}}$ respectively denote the bonding energy for Fe-N and X-N; n_{Fe} and n_{X} are the number of interstitial nitrogen respectively trapped at the Fe interstices and the X interstices; n is the total number of interstitial N atoms. **Eq. (S1)** immediately rationalizes the thermodynamic parameter $\Lambda = \Delta H^{\mathrm{mix}}_{\mathrm{X-N}} - \Delta H^{\mathrm{mix}}_{\mathrm{Fe-N}}$, since $\Delta\varepsilon_{\mathrm{N-X}}$ the bonding energy difference between Fe-N and X-N is quantitatively corelated with the individual mixing enthalpy because (*20*):

$$\Delta H^{\mathrm{mix}}_{\mathrm{Fe-N}} \simeq Z\left(\varepsilon_{\mathrm{Fe-N}} - \frac{\varepsilon_{\mathrm{Fe-Fe}}+\varepsilon_{\mathrm{N-N}}}{2}\right) \propto \varepsilon_{\mathrm{Fe-N}} \tag{S2}$$

and:

$$\Delta H^{\mathrm{mix}}_{\mathrm{X-N}} \simeq Z\left(\varepsilon_{\mathrm{X-N}} - \frac{\varepsilon_{\mathrm{X-X}}+\varepsilon_{\mathrm{N-N}}}{2}\right) \propto \varepsilon_{\mathrm{X-N}} \tag{S3}$$

Thus:

$$\Lambda = \Delta H^{\mathrm{mix}}_{\mathrm{X-N}} - \Delta H^{\mathrm{mix}}_{\mathrm{Fe-N}} \propto (\varepsilon_{\mathrm{X-N}} - \varepsilon_{\mathrm{Fe-N}}) = \Delta\varepsilon_{\mathrm{N-X}} \tag{S4}$$

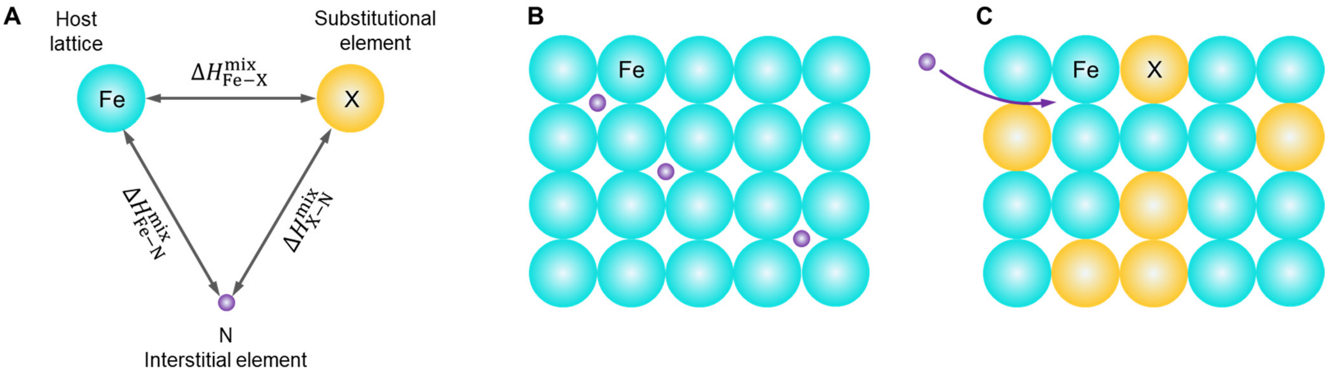

Fig. S14 | Theoretical considerations of N interstitial alloying in a pre-existing Fe-X substitutional alloy (A) Sketch of the energetics of the interactions considered. **(B)** The prototypical case of an Fe-N interstitial alloy. **(C)** Schematic of the interstitial alloying process conceived for the thermodynamic *treasure map* in main text **Fig. 1 C**.

By assuming the predominant contribution of configurational entropy change ($\mathcal{S}_{\mathrm{conf.}}$) over magnetic ($\mathcal{S}_{\mathrm{mag.}}$), phonon ($\mathcal{S}_{\mathrm{ph.}}$), or electronic ($\mathcal{S}_{\mathrm{el.}}$) entropy change during interstitial alloying and adopting the Boltzmann's entropy representation (*23*):

$$\mathcal{S}_{tot.} = \mathcal{S}_{\mathrm{mag.}} + \mathcal{S}_{\mathrm{ph.}} + \mathcal{S}_{\mathrm{el.}} + \mathcal{S}_{\mathrm{conf.}} \simeq \mathcal{S}_{\mathrm{conf.}} = k_B \ln(\Omega_{\mathrm{Fe-X-N}}) \tag{S5}$$

Where $\Omega_{\mathrm{Fe-X-N}}$ is the total number of possible interstitial solute microstates in an Fe-X-N ternary alloy, and can be calculated following:

$$\Omega_{\mathrm{Fe-X-N}} = \binom{n_{\mathrm{X}}}{\widetilde{N_{\mathrm{X}}}}\cdot\binom{n_{\mathrm{Fe}}}{\widetilde{N_{\mathrm{Fe}}}} = \frac{\widetilde{N_{\mathrm{X}}}!}{n_{\mathrm{X}}!(\widetilde{N_{\mathrm{X}}}-n_{\mathrm{X}})!}\cdot\frac{\widetilde{N_{\mathrm{Fe}}}!}{n_{\mathrm{Fe}}!(\widetilde{N_{\mathrm{Fe}}}-n_{\mathrm{Fe}})!} = \frac{\widetilde{N_{\mathrm{X}}}!}{n_{\mathrm{X}}!(\widetilde{N_{\mathrm{X}}}-n_{\mathrm{X}})!}\cdot\frac{\widetilde{N_{\mathrm{Fe}}}!}{(n-n_X)!(\widetilde{N_{Fe}}-n+n_X)!} \tag{S6}$$

In **Eq. (S6)**, $\widetilde{N_\mathrm{X}}$ and $\widetilde{N_\mathrm{Fe}}$ are the number of total available X and Fe interstices. Combining **Eqs. (S1)**, **(S5)**, and **(S6)**, the fully energetics of interstitial alloying can be expressed as:

$$\mathcal{G}_\mathrm{N-X} = n\varepsilon_\mathrm{Fe-N} + \Delta\varepsilon_\mathrm{N-X} n_\mathrm{X} - k_B \ln\left[\frac{\widetilde{N_\mathrm{X}}!}{n_\mathrm{X}!(\widetilde{N_\mathrm{X}}-n_\mathrm{X})!}\cdot\frac{\widetilde{N_\mathrm{Fe}}!}{(n-n_X)!(\widetilde{N_{Fe}}-n+n_X)!}\right] \quad \text{(S7)}$$

The thermodynamic equilibrium condition requires $\partial\mathcal{G}_\mathrm{N-X}/\partial n_\mathrm{X} = 0$, and $\partial^2\mathcal{G}_\mathrm{N-X}/\partial n_\mathrm{X}^2 > 0$:

$$\frac{n_\mathrm{X}}{n_\mathrm{Fe}} = \left(\frac{\widetilde{N_\mathrm{X}}-n_\mathrm{X}}{\widetilde{N_\mathrm{Fe}}-n_\mathrm{Fe}}\right)\exp\left(-\frac{\Delta\varepsilon_\mathrm{I-X}}{k_B T}\right) \simeq \frac{zc_\mathrm{X}}{1-zc_\mathrm{X}}\exp\left(-\frac{\Delta\varepsilon_\mathrm{I-X}}{k_B T}\right) \propto \frac{zc_\mathrm{X}}{1-zc_\mathrm{X}}\exp\left(-\frac{\Lambda}{k_B T}\right) \quad \text{(S8)}$$

In **Eq. (S8)**, z is the coordinate number, and $c_\mathrm{X} = N_\mathrm{X}/N$ is the molar fraction of substitutional alloying element. Re-shuffling **Eq. (S8)**, we arrive at an analytical solution under the Bragg-Williams mean-field approximation for the fraction of interstitial N trapped by substitutional solute X as a function of $\Delta\varepsilon_\mathrm{N-X}$, and hence the Λ parameter introduced in the present study:

$$f_\mathrm{I-X} = \frac{n_\mathrm{X}}{n} = \frac{n_\mathrm{X}}{n_\mathrm{Fe}}/\left(1+\frac{n_\mathrm{X}}{n_\mathrm{Fe}}\right) \simeq \frac{zc_\mathrm{X}\exp(-\Delta\varepsilon_\mathrm{I-X}/k_B T)}{1-zc_\mathrm{X}[1-\exp(-\Delta\varepsilon_\mathrm{I-X}/k_B T)]} \propto \frac{zc_\mathrm{X}\exp(-\Lambda/k_B T)}{1-zc_\mathrm{X}[1-\exp(-\Lambda/k_B T)]} \quad \text{(S9)}$$

Eq. (S9) thus unequivocally rationalizes the physical significance of the Λ parameter in a sense that it compares the tendency of forming strongly localized X-N or the prototypical Fe-N interaction pairs, as discussed in main text **Fig. 1 C**. For sanity check, we assign trial values to **Eq. (S9)** and reveal the physical trends that it motivates. We choose $c_\mathrm{X} = 0.1$, reflecting the 10 at.% substitutional alloying element content adopted in our synthesis (main text **Fig. 3** and **Supplementary Fig. S6**), $z = 4$ for the simplified two-dimensional simple cubic lattice, and assess how $f_\mathrm{I-X}$ changes over temperature at different $\Delta\varepsilon_\mathrm{I-X}$, and correspondingly, the different Λ values (**Fig. S15**).

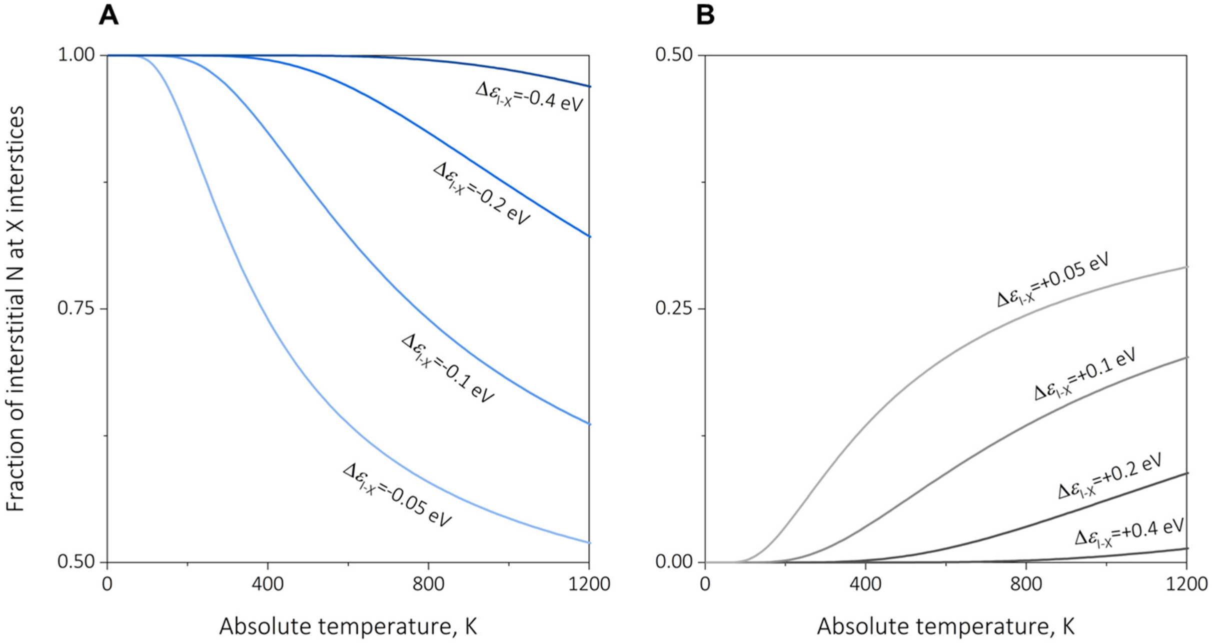

Fig. S15 | Theoretical temperature dependency of the $f_\mathrm{I-X}$ parameter with different $\Delta\varepsilon_\mathrm{N-X}$ values. (A) $\Delta\varepsilon_\mathrm{N-X} < 0$. **(B)** $\Delta\varepsilon_\mathrm{N-X} > 0$.

When $\Delta\varepsilon_\mathrm{N-X} < 0$ (correspondingly, $\Lambda < 0$), which hints at a significantly stronger X-N bond than the Fe-N bond, it is seen in **Fig. S15 A** that $f_\mathrm{I-X} \to 1.0$ as temperature decreases down to absolute 0 K, implying that all the N interstitial atoms will occupy

the X interstices (at the ground state), practically either forming local clusters or X-rich nitride phase. When temperature increases, $f_{\mathrm{I-X}}$ starts to decrease, which is due to the configurational entropy contribution outlined in **Eqs. (S5)-(S7).** The more negative $\Delta\varepsilon_{\mathrm{N-X}}$ is, the less pronounced attenuation is present in $f_{\mathrm{I-X}}$. These physical revelations further suggest that substitutional alloying elements showing $\Delta\varepsilon_{\mathrm{N-X}} < 0$ (and hence, $\Lambda < 0$) exhibit the tendencies to promote N interstitial solubility. This is unequivocally consistent with the trends seen in **Fig. S13 B** in which N solubility tends to increase when Cr and Mn serve as the substitutional alloying element in Fe, where the Λ value is respectively -20 and -32 kJ/mol. When $\Delta\varepsilon_{\mathrm{N-X}}$ becomes significantly negative, such as in the cases of Ti and V substitutional alloying in Fe (computed Λ is -103 and -56 kJ/mol), our model predicts that local clustering of Ti-N or V-N will be present, and this has been validated more recently via transmission electron microscopy observations (*18, 66*). *Vice versa*, a positive $\Delta\varepsilon_{\mathrm{N-X}}$ value (correspondingly $\Lambda > 0$, **Fig. S15 B**) suggests either the substitutional alloying element X tends to decrease N solubility, as evidenced in the case of Ni and Co in **Fig. S13 B** (Λ reaches +18 and +12 kJ/mol, respectively), or instead leads to Fe-N clustering. We note that in both cases, the increase in temperature always counteracts the local clustering trend (if any), largely due to the entropic contribution outlined in **Eq. (S6)**.

With all the foregoing theoretical considerations, we have physically rationalized the use of the thermodynamic parameter $\Lambda = \Delta H^{\mathrm{mix}}_{\mathrm{X-N}} - \Delta H^{\mathrm{mix}}_{\mathrm{Fe-N}}$ to assess the capability of N interstitial alloying, as outlined in the thermodynamic *treasure map* in main text **Fig. 1 C**. The analytical solution obtained in **Eq. (S9)** that follows the Bragg-Williams mean-field approach can also be directly combined with density functional theory-based simulations of the $\Delta\varepsilon_{\mathrm{N-X}}$ values of various Fe-X-N alloys, through which rigorously quantitative prediction of the interstitial configuration is possible. In view of the significance of bonding energy, we also propose a complementary thermodynamic *treasure map* that instead highlights electron negativity difference between the Fe-N and the X-N pairs, through: $K = \Delta\chi^2_{\mathrm{Fe-N}} - \Delta\chi^2_{\mathrm{X-N}}$, where χ denotes the electron negativity of a certain element, following the Allen scale. The resulting **Fig. S16** reveals desired consistency with main text **Fig. 1 C**, and a linear correlation is also present between the $K = \Delta\chi^2_{\mathrm{Fe-N}} - \Delta\chi^2_{\mathrm{X-N}}$ and the $\Lambda = \Delta H^{\mathrm{mix}}_{\mathrm{X-N}} - \Delta H^{\mathrm{mix}}_{\mathrm{Fe-N}}$ parameters.

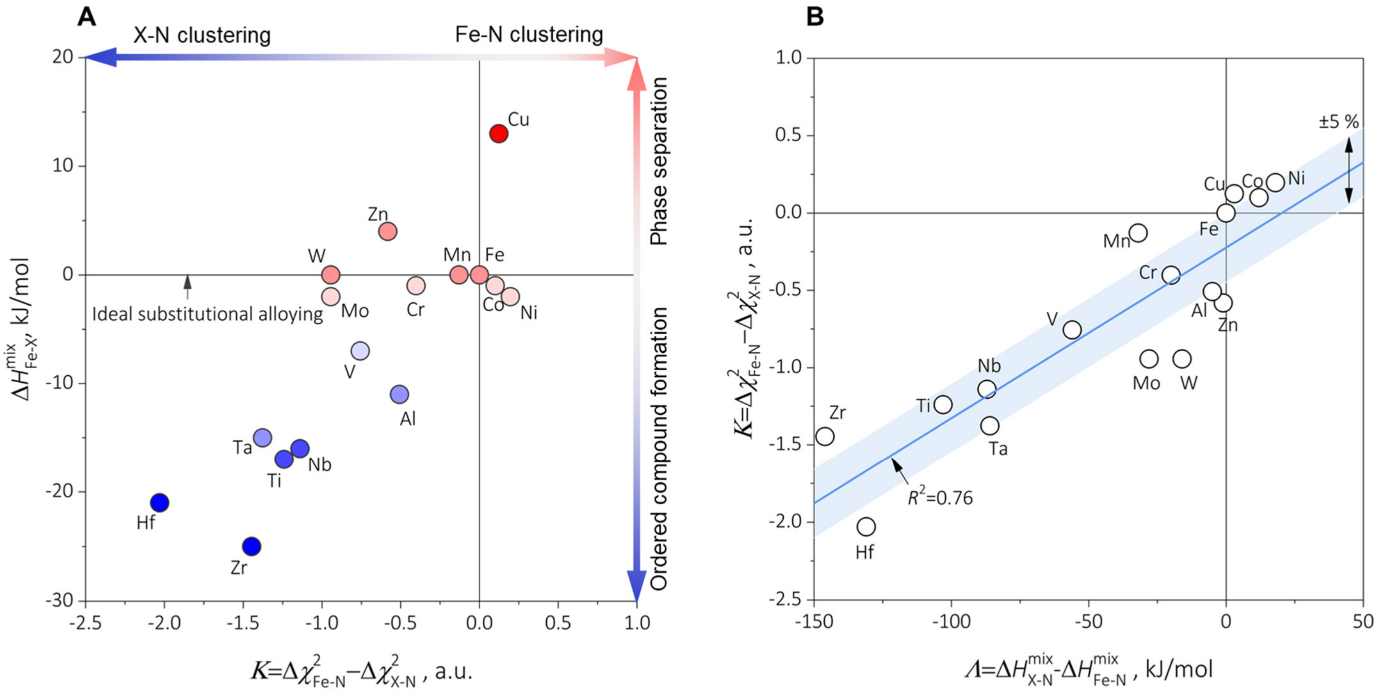

Fig. S16 | Complementary thermodynamic treasure map guiding interstitial alloying. **(A)** $\Delta H^{\mathrm{mix}}_{\mathrm{Fe-X}}$ versus $K = \Delta\chi^2_{\mathrm{Fe-N}} - \Delta\chi^2_{\mathrm{X-N}}$ for all the surveyed elements in main text **Fig. 1 C**. Here the electron negativity follows the Allen scale. **(B)** Correlation analysis between $K = \Delta\chi^2_{\mathrm{Fe-N}} - \Delta\chi^2_{\mathrm{X-N}}$ and $\Lambda = \Delta H^{\mathrm{mix}}_{\mathrm{X-N}} - \Delta H^{\mathrm{mix}}_{\mathrm{Fe-N}}$, in which most of elements locate in the ± 5 % error range of the fitted line.

Note 2. Supporting analyses for the *in situ* synchrotron X-ray observations

For clarifying the synthesis micro-mechanisms, we have revealed in main text **Fig. 5** *in situ* synchrotron X-ray results of the phase constitution change when Fe_2O_3 and NiO mixed oxides undergo reactive dealloying-alloying in NH_3. In this ***Note***, we provide further analyses supporting the main text argumentation that Ni substitutional alloying can drive transformations in the nitride phases.

We resort to the *in situ* SXRD results shown in main text **Fig. 5 A** and magnify here the diffraction patterns obtained in the 550-700 °C temperature range (**Fig. S17**). A non-monotonic diffraction pattern shift phenomenon is notable in the rhombohedron-structured Fe_3N phase: upon its formation, all the diffraction peaks firstly shift to the higher 2θ direction until ~600 °C, immediately followed by a sharp turn to the lower 2θ region. The same group of diffraction peaks again shift to the higher 2θ direction as temperature further increases up to 700 °C. This kind of non-monotonic shift, however, only leads to the relative position change in each reflection group; the global crystallographic symmetry, on the other hand, remains unperturbed. To better understand the underlying mechanisms, we have conducted a separate set of *in situ* measurement under the identical condition solely on pristine Fe_2O_3 with no NiO involved. Significant differences in the evolution of the Fe_3N diffraction patterns are clearly resolved: when such a phase forms from the pristine Fe_2O_3 oxide, all the diffraction patterns consistently shift to the higher 2θ region up to 700 °C. It is suggestive that Ni dissolution into the Fe_3N lattice is most likely to be responsible for the non-monotonic shift in the diffraction peaks seen in **Fig. S17 A**. We next provide quantitative analyses and discuss the plausible phase transformation micro-mechanisms, starting with the lattice constant change, as determined from Rietveld refinement.

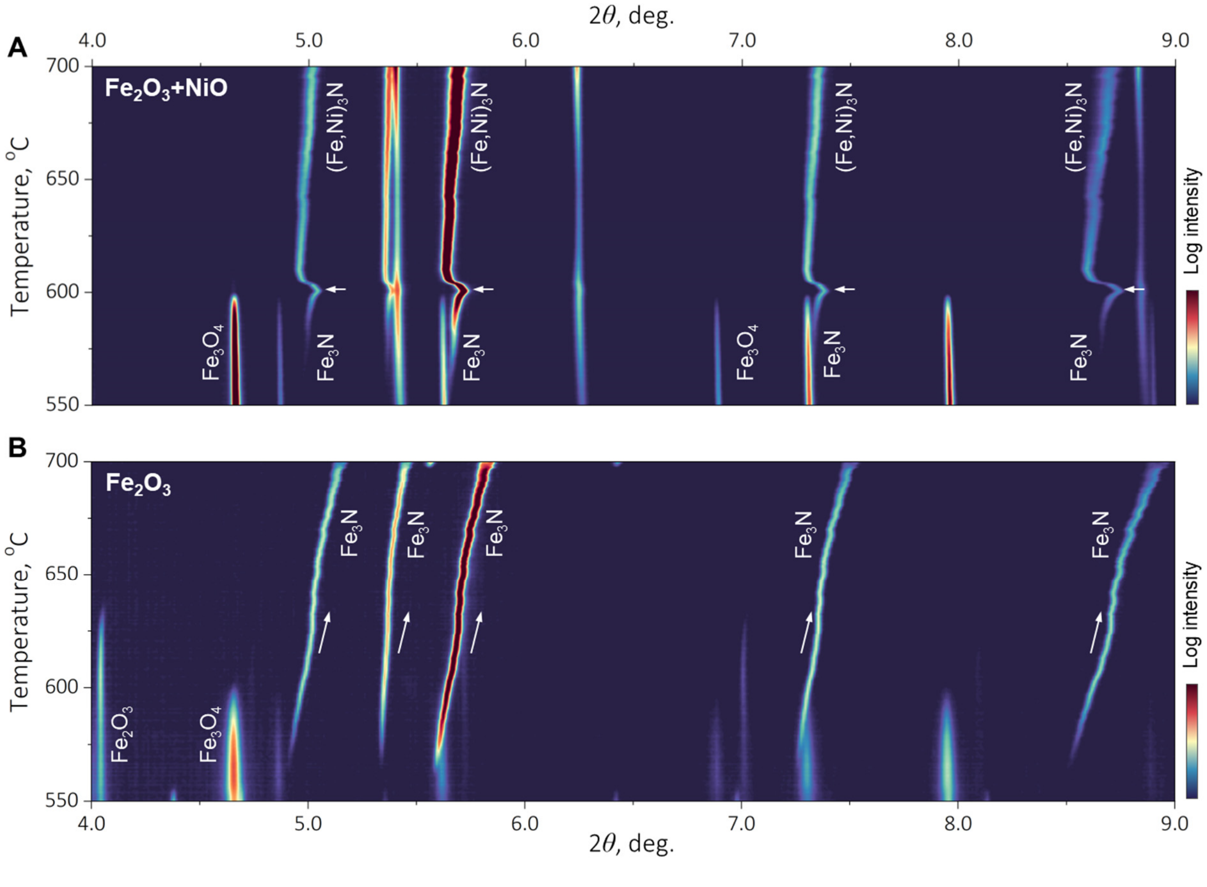

Fig. S17 | Comparison of *in situ* SXRD results obtained from the Fe_2O_3+NiO mixed oxides and the pristine Fe_2O_3. (A) Zoomed in view of the diffractograms acquired in the 550-700 °C temperature range for the Fe_2O_3+NiO mixed oxides. **(B)** *In situ* diffractograms of the Fe_3N phase in the same temperature for the pristine Fe_2O_3 oxide.

Fig. S18 compares the lattice constant evolution of the Fe_3N phase obtained from the Fe_2O_3+NiO mixed and the pristine Fe_2O_3 oxide. As temperature increases, the lattice constants a and c of the Fe_3N phase formed in the Fe_2O_3 case both exhibit monotonic decreasing trends, which signifies N loss, as well-documented in the literature (*67–69*). These two lattice constants of the same Fe_3N phase seen from the mixed oxide study exhibit stepwise evolution trends as a function of temperature: in the 570-600 ºC temperature range, both a and c consistently decrease as temperature increases, which is mostly owing to nitrogen loss. A stepwise jump incepts at ~600 ºC and finishes at ~607 ºC, leading to burst-like increase in a and c, respectively from 4.721 to 4.793 Å, and from 4.419 to 4.443 Å. After that, both a and c reveal much more moderate decreasing trends compared with the Fe3N phase obtained from Fe_2O_3. At 700 ºC, differences in a and c are significant in both cases, respectively achieving 0.154 and 0.078 Å. Since the lattice constants a and c directly reflects the molar volume (V_m) of the corresponding crystal, a thermodynamic response function in its nature (*70*): $V_m = (\partial G/\partial p)_T$, the observed stepwise jump thus unambiguously supports a discontinuity in V_m, which by definition, indicating a first-order phase transformation.

While detailed chemical composition of the nitride phases still call for extensive future APT work, the current *in situ* SXRD results imply the following plausible mechanism for the observed phase transformation: at the initial state, the Fe_3N phase formed in the mixed oxide exhibits similar chemical composition as in the pristine Fe_2O_3 case, and the decrease in the lattice constants can be rationalized by the nitrogen loss. Because of the reducing atmosphere, the NiO→Ni reaction is also being activated in parallel, leading to immense Ni substitution alloying in the prototypical Fe_3N phase. We also note that no metallic Ni phase can be seen beyond 550 ºC (**Supplementary Fig. S11**), suggesting its expedited dissolution into the nitride. Such a process is deemed significant and consequently activates a first-order phase transformation, forming an $(Fe,Ni)_3N$ phase, while the global crystallographic symmetry is still maintained. The new phase, however, appears to be thermodynamically more stable against N loss upon temperature increase, resulting in the significantly larger a and c observed at 700 ºC. For all these reasons, the involvement of Ni substitutional alloying is indispensable in driving transformations in the Fe_3N phase.

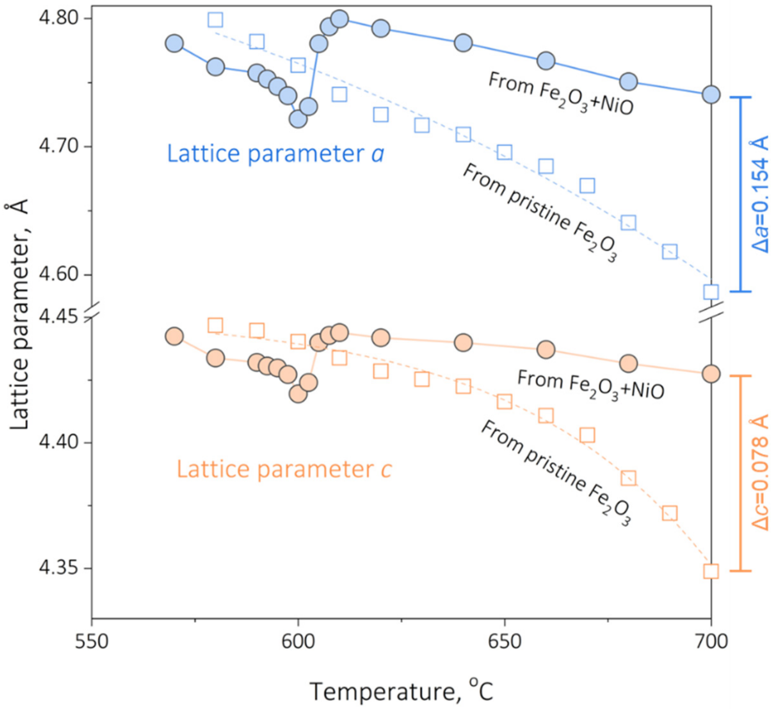

Fig. S18 | Comparison of the temperature dependency of the lattice constants a and c for the Fe_3N phase obtained from the Fe_2O_3+NiO mixed oxides and the pristine Fe_2O_3. All the experimental boundary conditions have been kept identical for both sets of *in situ* measurements.

Captions for the supplementary Videos

Video S1 | Three-dimensional visualization of the nano-structured porous Fe-Ni-N alloy

Video S2 | Three-dimensional visualization of the connected and the isolated pores

Video S3 | *In situ* synchrotron X-ray observations of the synthesis process

REFERENCES AND NOTES

1. G. Masing, Zur Theorie der Resistenzgrenzen in Mischkristallen. ["On the theory of resistivity thresholds in solid solution crystals."] *Zeitschrift für Anorg. und Allg. Chemie* **118**, 293–308 (1921).

2. J. Erlebacher, M. J. Aziz, A. Karma, N. Dimitrov, K. Sieradzki, Evolution of nanoporosity in dealloying. *Nature* **410**, 450–453 (2001).

3. C. Zhu, Z. Qi, V. A. Beck, M. Luneau, J. Lattimer, W. Chen, M. A. Worsley, J. Ye, E. B. Duoss, C. M. Spadaccini, C. M. Friend, J. Biener, Toward digitally controlled catalyst architectures: Hierarchical nanoporous gold via 3D printing. *Sci. Adv.* **4**, eaas9459 (2018).

4. Y. Li, B.-N. Ngo-Dinh, J. Markmann, J. Weissmüller, Evolution of length scales and of chemical heterogeneity during primary and secondary dealloying. *Acta Mater.* **222**, 117424 (2022).

5. J. Weissmüller, K. Sieradzki, Dealloyed nanoporous materials with interface-controlled behavior. *MRS Bull.* **43**, 14–19 (2018).

6. D. Raabe, The materials science behind sustainable metals and alloys. *Chem. Rev.* **123**, 2436–2608 (2023).

7. S. Wei, Y. Ma, D. Raabe, One step from oxides to sustainable bulk alloys. *Nature* **633**, 816–822 (2024).

8. L. Brewer, Thermodynamic properties of the oxides and their vaporization processes. *Chem. Rev.* **52**, 1–75 (1953).

9. B. C. H. Steele, Oxygen transport and exchange in oxide ceramics. *J. Power Sources* **49**, 1–14 (1994).

10. J. S. Lim, H.-H. Nahm, M. Campanini, J. Lee, Y.-J. Kim, H.-S. Park, J. Suh, J. Jung, Y. Yang, T. Y. Koo, M. D. Rossell, Y.-H. Kim, C.-H. Yang, Critical ionic transport across an oxygen-vacancy ordering transition. *Nat. Commun.* **13**, 5130 (2022).

11. Y. Dong, Redox enhanced slow ion kinetics in oxide ceramics. *J. Am. Ceram. Soc.* **107**, 1905–1916 (2024).

12. P. Cavaliere, L. Dijon, A. Laska, D. Koszelow, Hydrogen direct reduction and reoxidation behaviour of high-grade pellets. *Int. J. Hydrogen Energy* **49**, 1235–1254 (2024).

13. S.-H. Kim, X. Zhang, Y. Ma, I. R. Souza Filho, K. Schweinar, K. Angenendt, D. Vogel, L. T. Stephenson, A. A. El-Zoka, J. R. Mianroodi, M. Rohwerder, B. Gault, D. Raabe, Influence of microstructure and atomic-scale chemistry on the direct reduction of iron ore with hydrogen at 700°C. *Acta Mater.* **212**, 116933 (2021).

14. I. McCue, E. Benn, B. Gaskey, J. Erlebacher, Dealloying and dealloyed materials. *Annu. Rev. Mater. Res.* **46**, 263–286 (2016).

15. E. J. Mittemeijer, *Fundamentals of Materials Science* (Springer, 2021).

16. E. J. Mittemeijer, M. A. J. Somers, *Thermochemical Surface Engineering of Steels: Improving Materials Performance* (Elsevier, 2014).

17. N. Yasuda, Y. Mochizuki, N. Tsubouchi, T. Akiyama, Reduction and nitriding behavior of hematite with ammonia. *ISIJ Int.* **55**, 736–741 (2015).

18. T. Furuhara, Y. Zhang, M. Sato, G. Miyamoto, M. Enoki, H. Ohtani, T. Uesugi, H. Numakura, Sublattice alloy design of high-strength steels: Application of clustering and nanoscale precipitation of interstitial and substitutional solutes. *Scr. Mater.* **223**, 115063 (2023).

19. T. Fujita, P. Guan, K. McKenna, X. Lang, A. Hirata, L. Zhang, T. Tokunaga, S. Arai, Y. Yamamoto, N. Tanaka, Y. Ishikawa, N. Asao, Y. Yamamoto, J. Erlebacher, M. Chen, Atomic origins of the high catalytic activity of nanoporous gold. *Nat. Mater.* **11**, 775–780 (2012).

20. C. H. P. Lupis, *Chemical Thermodynamics of Materials* (Prentice Hall, 1993).

21. L. Holappa, M. Kekkonen, A. Jokilaakso, J. Koskinen, A review of circular economy prospects for stainless steelmaking slags. *J. Sustain. Metall.* **7**, 806–817 (2021).

22. Y. Ma, J. W. Bae, S. Kim, M. Jovičević-Klug, K. Li, D. Vogel, D. Ponge, M. Rohwerder, B. Gault, D. Raabe, Reducing iron oxide with ammonia: A sustainable path to green steel. *Adv. Sci.* **2300111**, 1–7 (2023).

23. R. K. Pathria, P. D. Beale, *Statistical Mechanics* (Elsevier, ed. 3, 2011).

24. D. G. Löffler, L. D. Schmidt, Kinetics of NH_3 decomposition on iron at high temperatures. *J. Catal.* **44**, 244–258 (1976).

25. E. R. Jette, F. Foote, Precision determination of lattice constants. *J. Chem. Phys.* **3**, 605–616 (1935).

26. M. Yousuf, P. C. Sahu, H. K. Jajoo, S. Rajagopalan, K. G. Rajan, Effect of magnetic transition on the lattice expansion of nickel. *J. Phys. F Met. Phys.* **16**, 373–380 (1986).

27. L. Vegard, Die Konstitution der Mischkristalle und die Raumfüllung der Atome. *Zeitschrift für Phys.* **5**, 17–26 (1921).

28. C. Soyarslan, S. Bargmann, M. Pradas, J. Weissmüller, 3D stochastic bicontinuous microstructures: Generation, topology and elasticity. *Acta Mater.* **149**, 326–340 (2018).

29. Z. Lu, C. Li, J. Han, F. Zhang, P. Liu, H. Wang, Z. Wang, C. Cheng, L. Chen, A. Hirata, T. Fujita, J. Erlebacher, M. Chen, Three-dimensional bicontinuous nanoporous materials by vapor phase dealloying. *Nat. Commun.* **9**, 276 (2018).

30. S. J. L. Kang, *Sintering: Densification, Grain Growth and Microstructure* (Elsevier, 2005).

31. A. J. Hallinan, A review of the Weibull distribution. *J. Qual. Technol.* **25**, 85–93 (1993).

32. E. Nes, N. Ryum, O. Hunderi, On the Zener Drag. *Acta Mater.* **33**, 11–22 (1985).

33. S. Zaefferer, N. N. Elhami, P. Konijnenberg, "Electron backscatter diffraction (EBSD) techniques for studying phase transformations in steels", in *Phase Transformations in Steels*, E. Pereloma, D. V. Edmonds, Eds. (Woodhead Publishing Limited, 2012).

34. J. H. van der Merwe, G. J. Shiflet, The role of structural ledges at phase boundaries-III. F.C.C.-B.C.C. interfaces in Kurdjumov-Sachs orientation. *Acta Metall. Mater.* **42**, 1199–1205 (1994).

35. M. S. Wechsler, On the theory of martensitic transformations. The generalized lattice invariant shear and the degeneracy of solutions for the cubic to tetragonal transformation. *Acta Metall.* **7**, 793–802 (1959).

36. A. Argon, *Strengthening Mechanisms in Crystal Plasticity* (Oxford Univ. Press, 2007), vol. 9780198516.

37. V. A. Lobodyuk, Y. Y. Meshkov, E. V. Pereloma, On tetragonality of the martensite crystal lattice in steels. *Metall. Mater. Trans. A Phys. Metall. Mater. Sci.* **50**, 97–103 (2019).

38. D. T. Keating, A. N. Goland, Atomic displacements in iron martensite. *Acta Metall.* **15**, 1805–1814 (1967).

39. A. Borgenstam, M. Hillert, Massive transformation in the Fe-Ni system. *Acta Mater.* **48**, 2765–2775 (2000).

40. T. B. Massalski, Massive transformations revisited. *Metall. Mater. Trans. A: Phys. Metall. Mater. Sci.* **33**, 2277–2283 (2002).

41. D. Raabe, M. Herbig, S. Sandlöbes, Y. Li, D. Tytko, M. Kuzmina, D. Ponge, P. P. Choi, Grain boundary segregation engineering in metallic alloys: A pathway to the design of interfaces. *Curr. Opin. Solid State Mater. Sci.* **18**, 253–261 (2014).

42. S. Wei, J. Kang, C. C. Tasan, An in situ synchrotron X-ray study of reverse austenitic transformation in a metastable FeMnCo alloy. *J. Mater. Res.* **38**, 281–296 (2022).

43. B. H. Toby, R. B. Von Dreele, GSAS-II: The genesis of a modern open-source all purpose crystallography software package. *J. Appl. Crystallogr.* **46**, 544–549 (2013).

44. S. Wei, K. S. Kim, J. Foltz, C. C. Tasan, Discovering pyramidal treasures: Multi-scale design of high strength–ductility titanium alloys. *Adv. Mater.* **36**, 1–9 (2024).

45. M. Pal, Random forest classifier for remote sensing classification. *Int. J. Remote Sens.* **26**, 217–222 (2005).

46. S. Berg, D. Kutra, T. Kroeger, C. N. Straehle, B. X. Kausler, C. Haubold, M. Schiegg, J. Ales, T. Beier, M. Rudy, K. Eren, J. I. Cervantes, B. Xu, F. Beuttenmueller, A. Wolny, C. Zhang, U. Koethe, F. A. Hamprecht, A. Kreshuk, ilastik: Interactive machine learning for (bio)image analysis. *Nat. Methods* **16**, 1226–1232 (2019).

47. T. M. Schwarz, E. Woods, M. P. Singh, X. Chen, C. Jung, L. S. Aota, K. Jang, M. Krämer, S.-H. Kim, I. McCarroll, B. Gault, In situ metallic coating of atom probe specimen for enhanced yield, performance, and increased field-of-view. *Microsc. Microanal.*, ozae006 (2024).

48. A. T. Dinsdale, SGTE data for pure elements. *Calphad* **15**, 317–425 (1991).

49. W. F. Gale, T. C. Totemeier, *Smithells Metals Reference Book* (Elsevier, 2003).

50. C. Cai, S. Wei, Z. Yin, J. Bai, W. Xie, Y. Li, F. Qin, Y. Su, D. Wang, Oxygen vacancy formation and uniformity of conductive filaments in Si-doped Ta_2O_5 RRAM. *Appl. Surf. Sci.* **560**, 149960 (2021).

51. R. Chatten, A. V. Chadwick, A. Rougier, P. J. D. Lindan, The oxygen vacancy in crystal phases of WO_3. *J. Phys. Chem. B* **109**, 3146–3156 (2005).

52. Y. Hinuma, T. Toyao, T. Kamachi, Z. Maeno, S. Takakusagi, S. Furukawa, I. Takigawa, K.-I. Shimizu, Density functional theory calculations of oxygen vacancy formation and subsequent molecular adsorption on oxide surfaces. *J. Phys. Chem. C* **122**, 29435–29444 (2018).

53. A. H. Heuer, T. Nakagawa, M. Z. Azar, D. B. Hovis, J. L. Smialek, B. Gleeson, N. D. M. Hine, H. Guhl, H.-S. Lee, P. Tangney, W. M. C. Foulkes, M. W. Finnis, On the growth of Al_2O_3 scales. *Acta Mater.* **61**, 6670–6683 (2013).

54. Y.-C. Zhang, L. Pan, J. Lu, J. Song, Z. Li, X. Zhang, L. Wang, J.-J. Zou, Unraveling the facet-dependent and oxygen vacancy role for ethylene hydrogenation on Co_3O_4 (110) surface: A DFT+U study. *Appl. Surf. Sci.* **401**, 241–247 (2017).

55. H. Jabraoui, M. D. Rouhani, C. Rossi, A. Esteve, First-principles investigation of CuO decomposition and its transformation into Cu_2 O. *Phys. Rev. Mater.* **6**, 096001 (2022).

56. A. J. R. Hensley, Y. Wang, J.-S. McEwen, The partial reduction of clean and doped α-Fe_2O_3(0001) from first principles. *Appl. Catal. A Gen.* **582**, 116989 (2019).

57. U. Aschauer, N. Vonrüti, N. A. Spaldin, Effect of epitaxial strain on cation and anion vacancy formation in MnO. *Phys. Rev. B* **92**, 054103 (2015).

58. W. B. Zhang, N. Yu, W. Y. Yu, B. Y. Tang, Stability and magnetism of vacancy in NiO: A GGA+U study. *Eur. Phys. J. B.* **64**, 153–158 (2008).

59. A. Takeuchi, A. Inoue, Classification of bulk metallic glasses by atomic size difference, heat of mixing and period of constituent elements and its application to characterization of the main alloying element. *Mater. Trans.* **46**, 2817–2829 (2005).

60. W. M. Haynes, *CRC Handbook of Chemistry and Physics Online* (CRC Press, ed. 95, 2014).

61. N. Saeidi, F. Ashrafizadeh, B. Niroumand, F. Barlat, EBSD study of damage mechanisms in a high-strength ferrite-martensite dual-phase steel. *J. Mater. Eng. Perform.* **24**, 53–58 (2015).

62. M. P. Kashchenko, V. P. Vereshchagin, Nucleation centers and wave schemes of martensite growth in iron alloys. *Sov. Phys. J.* **32**, 592–595 (1989).

63. M. Y. Gutkin, K. N. Mikaelyan, V. E. Verijenko, Heterogeneous nucleation of martensite near free surface. *Acta Mater.* **49**, 3811–3819 (2001).

64. H. J. Goldschmidt, *Interstitial Alloys* (Springer New York, 1967).

65. C. Ko, R. B. McLellan, Thermodynamics of ternary nitrogen austenites. *Acta Metall.* **31**, 1821–1827 (1983).

66. G. Miyamoto, Y. Tomio, H. Aota, K. Oh-Ishi, K. Hono, T. Furuhara, Precipitation of nanosized nitrides in plasma nitrided Fe-M (M = Al, Cr, Ti, V) alloys. *Mater. Sci. Technol.* **27**, 742–746 (2011).

67. J. C. Tseng, D. Gu, C. Pistidda, C. Horstmann, M. Dornheim, J. Ternieden, C. Weidenthaler, Tracking the active catalyst for iron-based ammonia decomposition by in situ synchrotron diffraction studies. *ChemCatChem* **10**, 4465–4472 (2018).

68. T. Steiner, E. J. Mittemeijer, Alloying element nitride development in ferritic fe-based materials upon nitriding: A review. *J. Mater. Eng. Perform.* **25**, 2091–2102 (2016).

69. M. A. J. Somers, N. M. Van Der Pers, D. Schalkoord, E. J. Mittemeijer, Dependence of the lattice parameter of {γ} iron nitride, Fe_4N_{1-x}, on nitrogen content; Accuracy of the nitrogen absorption data. *Metall. Trans. A* **20**, 1533–1539 (1989).

70. B. Fultz, *Phase Transitions in Materials* (Cambridge Univ. Press, 2020).
